# Psychiatric nurses versus psychiatrists and pharmacists 'knowledge on polypharmacy practices in psychiatry: An interprofessional mixed-methods exploration

**DOI:** 10.1371/journal.pone.0327104

**Published:** 2026-07-14

**Authors:** Amal I. Khalil, Alhanouf A. Almuhalbidi, Reema T. Almutairi, Shahad S. Almutairi, Hatun H. Alansari, Atheer S. Almarri, Shaima K. Alzahrani, Joud S. Alzahrani

**Affiliations:** 1 King Abdullah International Medical Research Center, Jeddah, Saudi Arabia; 2 King Saud bin Abdulaziz University for Health Sciences, College of Nursing, Jeddah, Saudi Arabia; 3 Ministry of National Guard Health Affairs (MNGHA); 4 Menoufyia University, Faculty of Nursing, Egypt; University of Toronto, CANADA

## Abstract

**Background:**

Polypharmacy is often crucial for managing complex and treatment-resistant psychiatric disorders, yet it carries risks such as adverse drug interactions, medication non-compliance, and suboptimal health outcomes. Interprofessional perspectives on polypharmacy significantly influence clinical decision-making and prescribing practices.

**Aim:**

This research evaluates healthcare providers’ knowledge and attitudes regarding psychiatric polypharmacy, comparing the views of psychiatric nurses, psychiatrists, and pharmacists. It also explores how these factors impact prescribing behaviors and interprofessional collaboration.

**Methods:**

A convergent mixed-methods approach was employed at the Erada Complex for Mental Health and Addiction in Jeddah, Saudi Arabia. The study involved 221 healthcare providers, including psychiatrists (n = 32), psychiatric nurses (n = 158), and pharmacists (n = 31). Quantitative data were collected using validated scales to assess knowledge and attitudes, while qualitative insights were gathered through open-ended responses and group discussions.

**Results:**

Knowledge levels varied among the professionals, with psychiatrists possessing the most comprehensive understanding (84.2 ± 11.0), followed by pharmacists (81.5 ± 10.0) and psychiatric nurses (79.5 ± 9.8). Attitudes toward polypharmacy also differed, with psychiatrists showing the most favorable views (3.79 ± 0.49), whereas nurses and pharmacists were more cautious due to concerns about adverse effects and medication burden. A significant positive correlation (r = 0.653, p < 0.05) was observed between knowledge and attitude scores. Sociodemographic factors, such as professional experience and confidence in medication management, influenced both knowledge and attitudes regarding medication management. Qualitative findings highlighted interprofessional tensions, with psychiatric nurses advocating for more conservative approaches, psychiatrists emphasizing clinical necessity, and pharmacists focusing on optimizing medication safety.

**Conclusion:**

Healthcare providers demonstrated varying levels of awareness and attitudes toward psychiatric polypharmacy, shaped by their professional roles and responsibilities. While psychiatrists were more accepting of polypharmacy, psychiatric nurses expressed concerns about patient burden, and pharmacists prioritized safety considerations. Enhancing interprofessional collaboration and ongoing education on polypharmacy practices are essential for improving patient outcomes.

## Introduction

Polypharmacy, defined in the general population as the concurrent use of five or more medications, is increasingly prevalent across healthcare settings. In psychiatry, polypharmacy typically refers to the use of two or more psychiatric medications simultaneously [1,2]. While sometimes clinically justified—such as in treatment-resistant psychosis—polypharmacy carries significant risks, including drug-drug interactions, increased side effects, and potentially inappropriate prescribing, which may compromise treatment adherence and patient outcomes [1,3–5].

Among psychiatric populations, antipsychotic polypharmacy (APP) is associated with higher rates of adverse events, increased healthcare costs, and uncertain long-term safety compared to monotherapy options such as clozapine, which remains underutilized despite its proven efficacy in treatment-resistant schizophrenia [6,7]. APP is often employed when monotherapy fails, particularly for managing aggression or negative symptoms, yet evidence supporting superior efficacy is inconsistent. Moreover, APP is linked to metabolic disturbances, weight gain, and extrapyramidal symptoms (EPS), highlighting the need for careful prescribing [2,8].

Globally, the prevalence of polypharmacy has increased since the 1970s, partly due to fragmented healthcare systems that create variations in prescribing practices [3,9–11]. Long-acting injectable (LAI) antipsychotics, including dual LAI regimens, show promising outcomes for treatment-resistant cases, but their implementation varies widely [12,13]. Despite the recognized risks, psychiatrists continue to initiate APP due to concerns about relapse and patient violence, with studies in Nigeria and Australia reporting that 47% were “extremely likely” and 36% “very likely” to prescribe APP under such circumstances [14]. Institutional culture, perceived patient risk, and stigma surrounding mental illness further influence prescribing behaviors [12,15].

In addition to psychiatrists, nurses and pharmacists are integral to medication management. Pharmacists’ knowledge and practices differ across sectors in Saudi Arabia, and they play a crucial role in reducing medication errors, supporting deprescribing initiatives, and improving patient safety [5,16–18]. Evidence suggests that multidisciplinary approaches to polypharmacy management can optimize patient outcomes and minimize risks [13,19].

This study is based on behavioral and cognitive theories that explain how knowledge, beliefs, and contextual factors influence prescribing behavior, including the Theory of Planned Behavior (Ajzen, [20]) that describes how attitudes, perceived control, and social norms can contribute to the likelihood of engaging in polypharmacy, the Health Belief Model (Rosenstock, [21]) that emphasizes how perceived benefits and risks influence decision-making around multiple medication use, Self-Efficacy Theory (Bandura, [22]) that explains how confidence in managing complex regimens can lead to more favorable attitudes toward polypharmacy, and Cognitive Dissonance Theory (Festinger, [23]) that describes the tension that providers feel when their awareness of the risks of polypharmacy are at odds with clinical or institutional pressure to prescribe multiple drugs. In addition, the Pharmacotherapeutic Complexity Framework [24] provides a systems-level approach to understanding how providers and patients manage complex psychiatric medication regimens. All these theories provide an integrated foundation for exploring how cognitive, behavioral, and contextual factors influence healthcare providers’ knowledge and attitudes toward polypharmacy in psychiatry.

Despite the widespread use of polypharmacy, research indicates significant gaps in healthcare providers’ knowledge and attitudes regarding its safe and effective use. Surveys reveal limited understanding of pharmacological interactions, inconsistent adherence to guidelines, and no global consensus on appropriate use [9,14,15]. Gender-specific considerations, particularly for women of childbearing potential, further complicate prescribing decisions [16–18].

Given these challenges, this study adopts a mixed-methods approach to comprehensively assess healthcare providers’ knowledge and attitudes toward polypharmacy in psychiatric settings. Integrating quantitative and qualitative data will provide nuanced insights into clinical reasoning, decision-making processes, and the factors that influence polypharmacy practices. Such an approach can inform targeted education, guideline development, and multidisciplinary strategies to improve patient care [25,26].

### Significance of the study

This research is significant due to the prevalent issue of polypharmacy and its potential negative consequences. Firstly, it addresses a research gap regarding healthcare providers’ understanding and attitudes towards psychiatric polypharmacy, an area with limited data despite involving high-risk patient groups. Secondly, understanding provider perspectives is essential for developing tailored educational programs, policies, and clinical guidelines aimed at improving medication safety, adherence, and patient outcomes. Lastly, by identifying factors influencing prescribing choices—such as experience, training, institutional culture, and views on mental illness—this study can help create interventions to reduce inappropriate polypharmacy and enhance psychiatric care.

### Study aim and research questions

Aim: To assess healthcare providers’ knowledge and attitudes regarding polypharmacy in psychiatric settings and explore the factors influencing prescribing behaviors and interprofessional collaboration.

#### Research Questions:.

- How knowledgeable are healthcare providers about psychiatric polypharmacy?
- What are the attitudes of healthcare providers towards polypharmacy in psychiatric treatment?
- What factors (e.g., experience, training, institutional culture) impact prescribing decisions in psychiatric polypharmacy?

#### Working Hypotheses:.

- H1 ≠ Healthcare providers with greater knowledge of polypharmacy are likely to exhibit more cautious attitudes towards prescribing multiple psychiatric medications.
- H 2 ≠ Negative perceptions of mental illness and insufficient training are associated with higher instances of inappropriate polypharmacy practices.

## Methodology

### Research design

This study employed a convergent mixed-methods design, in which quantitative and qualitative data were collected concurrently, analyzed separately, and subsequently integrated to provide a comprehensive understanding of polypharmacy practices in psychiatric care. While previous research has examined healthcare providers’ knowledge and attitudes regarding psychiatric polypharmacy, a mixed-methods approach is essential to:

1. Explore underlying reasons, clinical reasoning, and contextual factors influencing prescribing practices.
2. Enhance validity through triangulation, confirming findings across quantitative and qualitative datasets.
3. Examine cultural and institutional factors, such as stigma and organizational norms, which may affect prescribing decisions.
4. Inform the development of targeted interventions and policies to optimize medication management and patient outcomes.

Although previous research has offered insight into the knowledge and attitudes of healthcare providers regarding psychiatric polypharmacy, a mixed-methods design is required to explore the underlying reasons, clinical reasoning, and contextual factors that may contribute to prescribing practices [27]. The integration of qualitative and quantitative data also improves validity by triangulation to confirm findings, increasing the credibility and depth of the study [28]; permits the examination of contextual and cultural factors, such as stigma and institutional norms, that can influence prescribing decisions in psychiatric care [29]; and allows the development of targeted interventions and policies to better manage medications and ultimately patient outcomes [30].Therefore, the mixed-methods approach is not only appropriate but necessary to comprehensively address the research questions and contribute meaningful insights to the field.

The integration of multiple perspectives from psychiatric nurses, psychiatrists, and pharmacists, ensures a more nuanced understanding of polypharmacy practices, beyond what single-method studies can provide.

This study adhered to the Strengthening the Reporting of Observational Studies in Epidemiology (STROBE) guidelines for reporting observational studies [31]. Qualitative components were informed by the Consolidated Criteria for Reporting Qualitative Research (COREQ) [32], and sex and gender reporting followed the Sex and Gender Equity in Research (SAGER) guidelines [33]. A completed STROBE checklist is provided as a supplementary file to ensure transparency and methodological rigor.

### Setting

The study was conducted at the Erada Complex for Mental Health and Addiction in Jeddah, Saudi Arabia—a Ministry of Health facility serving patients from Jeddah and neighboring regions (Makkah, Taif, and Alkonfedah). The complex includes six inpatient wards, six outpatient clinics, a 24-hour emergency unit, and serves as a teaching hospital for healthcare trainees.

### Participants

The study included psychiatrists, psychiatric nurses, and pharmacists who were actively engaged in patient care and medication management. Participants were required to possess valid professional licenses and practical experience in prescribing, dispensing, or overseeing psychiatric medications. Although the initial recruitment strategy broadly considered other mental health professionals and psychiatric patients, these groups were excluded from the final analysis because of the study’s specific focus on medication-related decision-making among key prescribing and medication-management professionals. Patient participants were included only in the qualitative exploratory phase when relevant, provided that they were clinically stable, compliant with treatment, and capable of effective communication. Individuals exhibiting acute psychotic symptoms or significant communication challenges were excluded. Data collection was conducted from February 3–1^st^ of May 2025.

### Sampling and recruitment

Based on a total population of approximately 250 healthcare professionals, a minimum sample of 152 participants was required, calculated at a 95% confidence level with a 5% margin of error. Additionally, an a priori power analysis was conducted using G Power (version 3.1), assuming a medium effect size (f² = 0.15), an alpha level of 0.05, a power of 0.80, and up to 10 predictors in a multiple regression analysis, which indicated a minimum sample size of 118. The larger sample size (n = 152) was retained to ensure sufficient power and enhance generalizability.

A stratified purposive sampling approach ensured representation across the professional roles. For the qualitative phase, recruitment continued until thematic saturation was reached, meaning that no new themes emerged.

**Note on bias:** Although purposive sampling may introduce selection bias, it was essential to ensure that the participants had relevant expertise in psychiatric polypharmacy. This limitation is explicitly acknowledged and discussed in this manuscript.

**Fig 1:**
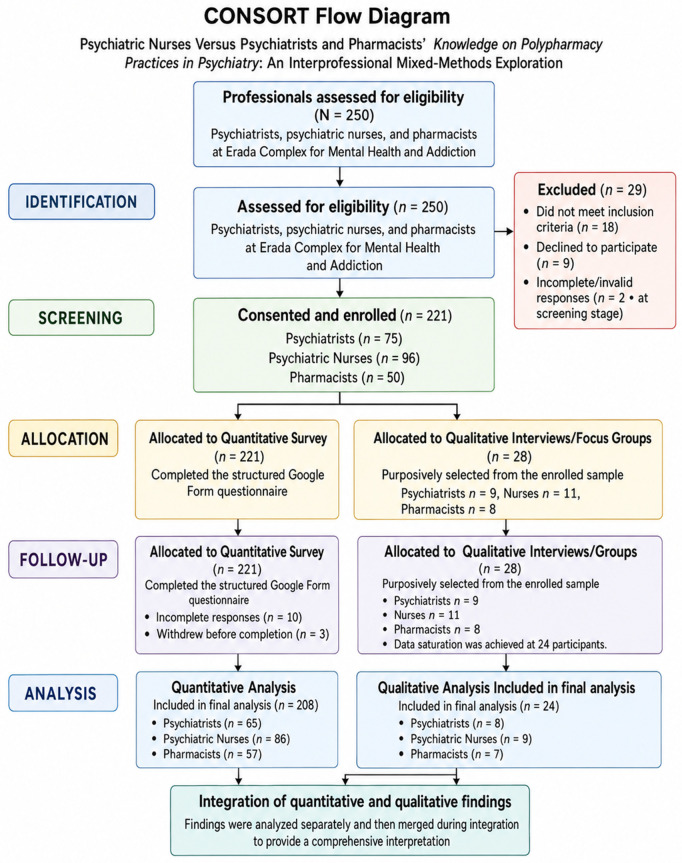
Consort flow diagram of sample recruitment N = 221.

A flow diagram illustrating participant recruitment, inclusion, and completion is shown in Fig 1. The consort flow diagram shows the purposive sampling technique used to recruit psychiatrists, nurses, and pharmacists with direct experience in psychiatric medication management. While this approach ensured the inclusion of knowledgeable participants, it may have introduced selection bias and limited generalizability. To mitigate this, efforts were made to include diverse healthcare professionals (psychiatrists, pharmacists, and nurses) with varying levels of experience and roles in the study. Nonetheless, the findings should be interpreted within the context of this non-probability sampling approach.

Recruitment was facilitated by the research team, in collaboration with the department heads, distributed study invitations to eligible participants. The inclusion criteria required at least six months of experience in psychiatric care. For the qualitative component, recruitment continued until **data saturation** was reached, meaning that no new themes emerged. The total number approached, those who consented, and those included in the final analysis will be reported to ensure transparency [9,10].

## Quantitative data collection

After obtaining ethical approval from the King Abdullah International Medical Research Center (KAIMRC) and the Institutional Review Board (IRB No. A02115) from Ministry of Health, data were collected through a validated, structured Google Form questionnaire distributed electronically to psychiatrists, nurses, and pharmacists.

### Self-reporting and bias mitigation

A major limitation of the knowledge assessment is its reliance on participants’ self-reported knowledge, which is inherently subjective and may not fully reflect actual prescription practices. To mitigate this limitation, this study incorporated a qualitative component alongside the quantitative survey. Semi-structured interviews allowed participants to describe their clinical reasoning, decision-making processes, and real-world experiences in managing psychiatric polypharmacy, and the integration of quantitative and qualitative findings provided a more comprehensive understanding of polypharmacy practices. While quantitative results indicated high knowledge and generally positive attitudes, qualitative data clarified the reasons behind these patterns. For example, pharmacists demonstrated moderately high knowledge scores and cautious yet supportive attitudes toward polypharmacy in the quantitative analysis, which was explained qualitatively by their emphasis on medication safety, drug interaction monitoring, and participation in multidisciplinary discussions. Similarly, although psychiatric nurses had comparatively lower knowledge scores, qualitative findings revealed that this was linked to limited exposure to prescribing guidelines and pharmacological decision-making rather than a lack of competence, as many nurses emphasized their need for continuous training and their primary role in medication administration and patient monitoring. In addition, the positive attitudes observed among frequent prescribers were contextualized by qualitative insights, indicating that such attitudes were driven by experience with complex, treatment-resistant cases requiring combination therapy. This triangulation demonstrates how qualitative themes directly inform the interpretation of quantitative scores, thereby enhancing the validity and depth of the findings [10,33].

While qualitative insights cannot eliminate self-report bias, they enhance the credibility and interpretive accuracy of the findings by revealing practical and nuanced information that surveys alone cannot capture. Additionally, anonymity was maintained in the survey responses to reduce social desirability bias.

## Quantitative data collection procedure

To achieve the study objectives, quantitative data were collected using three structured tools:

I. **A demographic questionnaire** designed to collect information from healthcare providers regarding age, gender, years of professional experience, education level, marital status, and prior training in polypharmacy.
II. **Polypharmacy Knowledge Assessment Tool (PKAT): Development and Validation**

The principal investigator (Khalil A.) developed a self-administered Polypharmacy Knowledge Assessment Tool (PKAT) to measure the knowledge of healthcare providers on polypharmacy in psychiatric practice. The instrument was developed according to guidelines for instrument construction and psychometric validation [32,33].

Development of the Instrument: An extensive literature review of clinical guidelines on polypharmacy published by the American Psychiatric Association (APA) and the National Institute for Health and Care Excellence (NICE), psychopharmacology education, medication safety, and interprofessional collaboration provided the basis for the item generation [9,10,34].A total of 30 items were developed that addressed the following knowledge domains: 1.Definition and general understanding of polypharmacy 2.Risks and complications of polypharmacy 3.Monitoring and clinical management strategies 4.Adherence to psychiatric prescribing guidelines

### Content validation

Content validity was established through review of the preliminary draft by a panel of five experts (two psychiatric nursing professors, two psychiatrists, and one clinical pharmacist), who rated each item for clarity, relevance, and representativeness using a four-point scale, with a mean Content Validity Index (CVI) of 0.92, indicating excellent agreement among reviewers [35]. The instrument was further refined and reduced to 25 items based on expert feedback to improve clarity and conceptual alignment.

### Translation and pilot testing

The tool was then translated into Arabic using a forward–backward translation process to maintain linguistic and conceptual equivalence. A pilot study was conducted with 15 healthcare providers to assess the clarity, comprehension, and cultural appropriateness of the items, and minor revisions were made based on feedback to improve readability and face validity.

### Construct validity and reliability

Exploratory Factor Analysis (EFA) with principal component extraction and varimax rotation was conducted to test construct validity, which indicated a four-factor solution (67.8% of total variance) consistent with conceptual domains identified during development, and Cronbach’s alpha was 0.91 (excellent reliability), with subscale alphas ranging from 0.81 to 0.89 (strong internal consistency) [36]. However, confirmatory factor analysis (CFA) was not performed; therefore, the factor structure should be interpreted with caution, and further validation is warranted.

### Scoring and interpretation

The final version of the PKAT consists of 25 items divided into the five domains mentioned above. Total scores are converted to a 0–100 scale and are interpreted as follows: •0–25: Low knowledge — further education strongly recommended•26–50: Moderate knowledge — basic understanding present, improvement needed•51–75: High knowledge — strong awareness of polypharmacy principles•76–100: Excellent knowledge — well-versed in clinical application of polypharmacy practices

**On summary** The PKAT underwent extensive development, expert review, translation, pilot testing, and psychometric evaluation and demonstrated strong content and construct validity and excellent internal consistency, thereby demonstrating that it is a reliable and valid tool for measuring the knowledge of healthcare providers on psychiatric polypharmacy in clinical and research contexts**.**

III. The Multidimensional Attitudes Toward Polypharmacy Scale (MAPS) developed by Köhler et al. in 2018 [36], provides a structured framework for assessing healthcare providers’ perspectives on polypharmacy. This scale evaluates multiple dimensions, including perceived necessity, concerns about adverse effects, and attitudes toward deprescribing, offering valuable insights into prescribing behaviors and medication management strategies. The scale is a 15-item instrument designed to evaluate healthcare providers’ attitudes toward polypharmacy. It assesses three key dimensions: (1) Perceived Necessity of Polypharmacy, which measures the belief that multiple medications are essential for effective treatment; (2) Concerns About Polypharmacy, capturing worries about potential risks like adverse drug interactions; and (3) Willingness to Deprescribe, reflecting openness to reducing or stopping unnecessary medications. Responses are rated on a 5-point Likert scale from 1 (Strongly Disagree) to 5 (Strongly Agree). Higher scores indicate stronger beliefs in the importance of polypharmacy or, in the case of concerns, greater apprehension about its risks. The MAPS has demonstrated strong psychometric properties, including good internal consistency, with reliability coefficients (Cronbach’s alpha) reported above 0.80, and solid construct validity, making it a reliable and valid tool for assessing healthcare providers’ views on polypharmacy.

### MAPS translation & cultural adaptation

The MAPS instrument underwent translation and cultural adaptation using standard forward–backward translation methods in Brazil. Initially, two independent bilingual experts translated the original English version into Arabic. This was followed by creating a reconciled version, which was then back-translated into English by two independent translators who were unaware of the original instrument. An expert panel, including specialists in psychiatry, mental health nursing, and clinical pharmacy, reviewed the back-translated version to ensure that it maintained semantic, conceptual, and content equivalence. Cultural adaptation was evaluated through expert review and pilot testing with a small group of healthcare professionals (n = 15) from the target population. Feedback was gathered on the clarity, relevance, and cultural appropriateness of each item. Minor adjustments were made to ensure contextual suitability without changing the original meanings of the items. This process confirmed the validity and reliability of the adapted instrument for use in the Saudi psychiatric setting.

### Data collection procedure

1. **Quantitative Data Collection:**
  - After receiving approvals from KAIMRC, the Ministry of Health IRB, and the Erada complex management, validated attitude and knowledge scales were converted into a Google Form and distributed electronically to healthcare providers (nurses, pharmacists, and physicians) working at the Erada complex for addiction and mental health.
  - AThe Google Form collected demographic information and responses to standardized knowledge and attitude scales regarding polypharmacy, including open-ended questions for qualitative insights.
  - ADistribution was managed to maximize participation, and reminders were sent to ensure adequate response rates. The recruitment of participants and data collection process for this study began on 03/02/2025 and ended on **01**/05/2025.
2. **Qualitative Data Collection Procedure Discussion Guide:**

The interviews examined healthcare providers’ views on polypharmacy, including their understanding of it, their confidence in prescribing multiple medications, and related clinical issues. Participants talked about the benefits and risks of polypharmacy, how they explain treatment plans, times when patients resisted, and what training they need. The interviews also covered teamwork between professionals and gathered ideas for improving the system.

A pilot focus group interview was held with three participants, one from each professional group in the study: nurses, physicians, and pharmacists. This assessed whether the discussion guide was clear, relevant, and useful. After improvements, four more focus group discussions were held using the updated guide. Participants were recruited in accordance with ethical guidelines, and recruitment continued until no new themes emerged. To fit participants’ schedules and preferences, semi-structured individual interviews were arranged. Nurses were interviewed in private rooms at their workplace. Physicians and pharmacists joined via Zoom. Each session began with ice-breaker activities, followed by open-ended questions about healthcare providers’ experiences, attitudes, challenges, and teamwork in managing polypharmacy.

Researchers used active listening and follow-up questions to help participants share detailed answers and explore their views more deeply. Each interview lasted 30–60 minutes and ended with a summary to ensure participants’ responses were accurate and complete. The interview guide had four parts: warming up, which explained the study and obtained consent; engagement, with introductory and ice-breaker questions; exploration, covering attitudes, challenges, and working with other professionals; and wrapping up, with a summary and closing remarks.

The interview guide had several steps. It began with a warm-up to explain the study’s purpose and collect both verbal and written consent. Next came questions to engage participants, followed by deeper questions about attitudes, challenges, and teamwork. The interview concluded with a summary and an opportunity for participants to share any final thoughts. All interviews were audio recorded and stored securely. Researchers also took notes on the setting and nonverbal cues.

The study used several methods to make sure the results were reliable and trustworthy. Credibility was improved by spending more time with participants, using different sources of information, and checking findings with participants. Detailed descriptions of the setting and participants helped others see if the results apply elsewhere. The research process was carefully documented to ensure dependability, and researchers kept journals to reduce bias and support confirmability.

### Ethical considerations

Ethical approval for the study was formally secured from the Institutional Review Board (IRB) at King Abdullah International Medical Research Center (KAIMRC), part of the National Guard Health Affairs in Jeddah, Saudi Arabia (Ref. No: A02115). Further authorization was obtained from the Research Unit at the College of Nursing in Jeddah, and permission to carry out the research was granted by the Saudi Arabian Ministry of Health and the administration of the Erada Complex for Addiction and Mental Health Hospital. Before participating, all individuals were thoroughly informed about the study’s aims, procedures, and their rights. Participation was voluntary, and participants were informed that they could opt out at any time without repercussions. Informed consent was obtained from all participants, both in writing and verbally, prior to data collection. For those who completed the survey online, consent was recorded via a secure digital form, while participants interviewed face-to-face signed written consent forms in the researcher’s presence. Anonymity and confidentiality were rigorously upheld; no personal identifiers were collected, and all data were securely stored, accessible only to the research team. The study adhered to the Declaration of Helsinki (2013 revision) and followed the ethical guidelines set by the Saudi Ministry of Health and KAIMRC for research involving human subjects.

### Data management and analysis plan

Data analysis incorporated both quantitative and qualitative methods. SPSS statistical software version 29.0 was used for the analysis. Categorical variables were described using frequencies and percentages, and continuous variables, such as knowledge and attitude scores regarding polypharmacy, were summarized using means and standard deviations. The Pearson correlation coefficient was used to assess the relationship between the knowledge and attitude scores. A multivariate analysis of variance (MANOVA) was performed to identify the factors influencing knowledge and attitudes toward polypharmacy. Significant predictors of overall knowledge and attitudes were examined using binary logistic regression. Statistical significance was set at p ≤ 0.05.

The quantitative aspects of the study were documented in accordance with the Strengthening the Reporting of Observational Studies in Epidemiology (STROBE) [37] guidelines, ensuring transparency, methodological rigor, and comprehensive reporting in observational research. For the qualitative aspect, thematic analysis was conducted using Braun and Clarke’s [38] six-phase framework, which involves familiarizing oneself with the data, generating initial codes, searching for themes, reviewing themes, defining and naming themes, and producing the final report. An inductive coding approach was used, with two researchers independently conducting the coding to enhance the credibility and minimize bias.

To ensure rigor and transparency in qualitative reporting, this study adhered to the Consolidated Criteria for Reporting Qualitative Research (COREQ) [39] guidelines. These standards guided the reporting of the research team’s characteristics, study design, data analysis procedures, and findings to ensure methodological trustworthiness. A coding framework (codebook) was developed to guide thematic analysis, incorporating both inductive codes derived from the data and concept-driven categories aligned with the study objectives and theoretical framework. Codes were organized into themes and subthemes reflecting healthcare providers’ knowledge, attitudes, decision-making processes, professional roles, and system-level influences on polypharmacy. The coding framework was iteratively refined through discussion between two independent researchers to ensure consistency and reliability. A summary of the coding framework is provided in (Supplementary S File [5].

The qualitative themes centered on healthcare providers’ attitudes, clinical experiences, and perceived challenges related to polypharmacy in psychiatric settings. During the interpretation phase, quantitative and qualitative findings were integrated using a mixed-methods triangulation approach. The quantitative results (knowledge and attitude scores) were aligned with the qualitative themes to provide a more comprehensive understanding of prescribing behaviors, decision-making processes, and interprofessional differences in psychiatric polypharmacy.

## Results

### Quantitative part

**Table 1** summarizes the demographic characteristics of the 221 healthcare providers in the study. The participants are relatively young, with a mean age of 35.9 years (SD = 7.7), and predominantly male (60.2%). Psychiatric nurses constitute the largest professional group (71.5%), followed by psychiatrists (14.5%) and pharmacists (14.0%). Regarding psychiatric experience, 36.2% have over 10 years, 27.6% have 6–10 years, 23.5% have 1–5 years, and 12.7% have less than 1 year. Most work in inpatient male psychiatric units (41.2%), outpatient mental health care (20.8%), or inpatient female psychiatric units (11.8%). Educationally, nearly half hold diplomas (47.5%), 39.8% have bachelor’s degrees, and smaller proportions hold master’s (6.8%) or PhD degrees (5.9%). Most (71.5%) lack additional psychopharmacology training, though the majority feel confident managing polypharmacy—43.9% very confident and 38.5% somewhat confident. A large majority frequently prescribe multiple medications, with 38% always and 45.7% often doing so. Most providers primarily care for adults (87.8%), with fewer focusing on geriatrics (6.8%), adolescents (3.6%), or children (1.8%). Overall, the cohort is experienced, mostly male psychiatric nurses focused on adult care, with many lacking specialized psychopharmacologies training but generally confident in their prescribing practices, which often involve polypharmacy consistent with their patient population’s needs

**Table 1:** distribution of the participants according to their Demographic data (N = 221).

Variable	N = 221
Age	35.9 ± 7.7
Gender	
*Female*	88 (39.8)
*Male*	133 (60.2)
Professional role	
*Psychiatrist*	32 (14.5)
*Psychiatrist nurse*	158 (71.5)
*Pharmacist*	31 (14.0)
Years of experience in psychiatry	
*Less than 1 year*	28 (12.7)
*1-5 years*	52 (23.5)
*6-10 years*	61 (27.6)
*More than 10 years*	80 (36.2)
Work setting	
*Inpatient psychiatric unit-male*	91 (41.2)
*Inpatient psychiatric unit-female*	26 (11.8)
*Outpatient mental health care*	46 (20.8)
*ER*	40 (18.1)
*Other*	18 (8.1)
Level of education	
*Diploma*	105 (47.5)
*Bachelor*	88 (39.8)
*Master*	15 (6.8)
*PhD*	13 (5.9)
Additional training in psychopharmacology	
*Yes*	63 (28.5)
*No*	158 (71.5)
Managing polypharmacy confidence	
*Very confident*	97 (43.9)
*Somewhat confident*	85 (38.5)
*Neutral*	27 (12.2)
*Somewhat unconfident*	12 (5.4)
*Very unconfident*	0 (0)
Multiple medication prescriptions	
*Always*	84 (38.0)
*Often*	101 (45.7)
*Sometimes*	29 (13.1)
*Rarely*	6 (2.7)
*Never*	1 (0.5)
Your primary population	
*Adults*	194 (87.8)
*Adolescents*	8 (3.6)
*Children*	4 (1.8)
*Geriatrics*	15 (6.8)

**Table 2** shows that psychiatrists achieved the highest knowledge scores, averaging 84.2 ± 11.0, with pharmacists scoring 81.5 ± 10.0 and psychiatric nurses at 79.5 ± 9.8. This difference was statistically significant (p = 0.044). In terms of attitude scores, psychiatrists again led with an average of 3.79 ± 0.49, followed by pharmacists at 3.69 ± 0.48 and psychiatric nurses at 3.63 ± 0.49, though this variation was not statistically significant (p = 0.367). These findings underscore a notable disparity in knowledge levels among different roles, especially highlighting the gap for psychiatric nurses.

**Table 2:** Differences in total knowledge and attitude scores toward polypharmacy in psychiatry by professional role (N = 221).

Scale	Psychiatrist	Pharmacist	Psychiatrist nurse	P-value
Knowledge	84.2 ± 11.0	81.5 ± 10.0	79.5 ± 9.8	0.044
Attitude	3.79 ± 0.49	3.69 ± 0.48	3.63 ± 0.49	0.367

**Mean± SD of scores**

**Fig 2:**
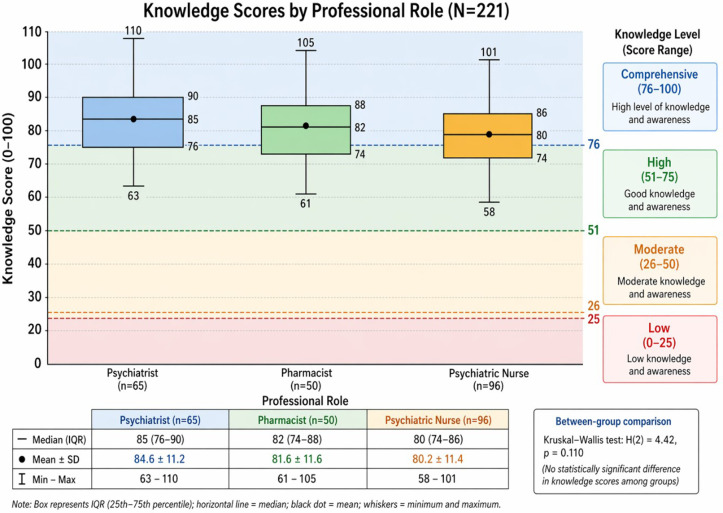
Total knowledge score about polypharmacy among health care professionals.

Fig 2 displays the enhanced box plots, which incorporate knowledge level thresholds and depict the distribution of knowledge scores on polypharmacy among the different professional groups. Overall, participants mostly displayed high to comprehensive knowledge levels, with most scores concentrated above the 75-point mark. Psychiatrists had the highest central tendency (median = 85, mean = 84.6 ± 11.2), followed by pharmacists (median = 82, mean = 81.6 ± 11.6) and psychiatric nurses (median = 80, mean = 80.2 ± 11.4). Despite these differences, the interquartile ranges showed significant overlap across all groups, suggesting that the distribution of the knowledge scores was broadly similar.

The variability in the scores within each group was moderate, with no extreme outliers significantly affecting the overall trend. The distribution of knowledge-level bands showed that most participants were in the “comprehensive” range (76–100), a smaller number were in the “high” range (51–75), and none were in the low or moderate ranges. An inferential analysis using the Kruskal–Wallis’s test found no statistically significant differences in knowledge scores among psychiatrists, pharmacists, and psychiatric nurses (H (2) = 4.42, p = 0.110), suggesting similar knowledge levels across these professional groups.

**Table 3** illustrates the understanding of polypharmacy in psychiatry among psychiatrists, nurses, and pharmacists (N = 221). On average, scores are high (3.7–4.45), suggesting a solid grasp of polypharmacy concepts, associated risks, and management strategies across these professional groups. Psychiatrists typically achieved slightly higher scores than nurses and pharmacists, this difference was statistically significant (p = 0.044), indicating variability in knowledge levels across professional groups. The highest scores were recorded for items focusing on regular medication reviews, patient involvement, and side effect reporting, indicating a strong acknowledgment of monitoring and patient-centered practices. Conversely, lower scores were found in areas like cognitive impairment risks, knowledge of deprescribing strategies, and awareness of clinical guidelines, pointing to possible knowledge gaps. The overall similarity in scores among the roles (p > 0.05 for most items) suggests consistent knowledge levels, while also highlighting the need for targeted continuing education and improved interprofessional collaboration to enhance polypharmacy management in psychiatric care. Given the significant overall difference observed for Item #1 (understanding of polypharmacy), Bonferroni-adjusted post-hoc pairwise comparisons were conducted. The results indicated that psychiatrists had significantly higher scores than psychiatric nurses (p < 0.05), while no statistically significant differences were found between psychiatrists and pharmacists or between pharmacists and nurses.

**Table 3:** Knowledge scale of polypharmacy in psychiatry by professional group (N = 221).

Statement	Psychiatrists Mean ± SD (%)	Nurses Mean ± SD (%)	Pharmacists Mean ± SD (%)	p-value
I understand the term “polypharmacy” as the use of multiple medications by a patient, particularly in psychiatric treatment	4.20 ± 0.70 (84%)	4.05 ± 0.80 (81%)	3.95 ± 0.85 (79%)	0.04*
I am aware of the potential risks associated with polypharmacy in psychiatric patients	4.15 ± 0.75 (83%)	4.00 ± 0.80 (80%)	4.10 ± 0.70 (82%)	0.12
Polypharmacy is often necessary in psychiatric practice to manage complex mental health conditions	4.10 ± 0.80 (82%)	3.95 ± 0.90 (79%)	4.05 ± 0.85 (81%)	0.08
I can differentiate between necessary polypharmacy and inappropriate medication use	4.05 ± 0.70 (81%)	3.95 ± 0.75 (79%)	4.00 ± 0.80 (80%)	0.10
I am familiar with the criteria for evaluating appropriate polypharmacy in psychiatry	3.95 ± 0.80 (79%)	3.85 ± 0.85 (77%)	3.90 ± 0.80 (78%)	0.15
Polypharmacy increases the likelihood of drug-drug interactions in psychiatric patients	4.15 ± 0.70 (83%)	4.05 ± 0.75 (81%)	4.10 ± 0.70 (82%)	0.20
I understand the potential for increased side effects with polypharmacy in psychiatric treatment	4.20 ± 0.75 (84%)	4.10 ± 0.80 (82%)	4.10 ± 0.75 (82%)	0.25
Patients on polypharmacy are more likely to experience medication non-adherence	4.05 ± 0.80 (81%)	4.00 ± 0.85 (80%)	4.05 ± 0.80 (81%)	0.30
Polypharmacy can contribute to cognitive impairment in psychiatric patients	3.80 ± 0.85 (76%)	3.70 ± 0.90 (74%)	3.70 ± 0.85 (74%)	0.28
I am aware of how polypharmacy can exacerbate comorbid physical health conditions in psychiatric patients	3.95 ± 0.90 (79%)	3.85 ± 0.95 (77%)	3.80 ± 0.90 (76%)	0.22
Regular medication reviews are crucial for managing polypharmacy in psychiatric care	4.45 ± 0.65 (89%)	4.35 ± 0.70 (87%)	4.35 ± 0.65 (87%)	0.15
I know how to assess the effectiveness and necessity of each medication in a patient’s polypharmacy regimen	4.00 ± 0.80 (80%)	3.90 ± 0.85 (78%)	3.85 ± 0.85 (77%)	0.18
I am familiar with deprescribing strategies to reduce polypharmacy when appropriate	3.85 ± 0.90 (77%)	3.75 ± 0.95 (75%)	3.80 ± 0.90 (76%)	0.20
I understand the importance of involving patients in discussions about their polypharmacy treatment plans	4.25 ± 0.70 (85%)	4.15 ± 0.75 (83%)	4.15 ± 0.70 (83%)	0.12
I monitor for signs of adverse drug reactions or toxicity in psychiatric patients on multiple medications	4.10 ± 0.75 (82%)	4.05 ± 0.80 (81%)	4.05 ± 0.75 (81%)	0.25
I am aware of clinical guidelines that provide recommendations for managing polypharmacy in psychiatry	3.85 ± 0.90 (77%)	3.75 ± 0.95 (75%)	3.80 ± 0.90 (76%)	0.22
Polypharmacy should always be considered a last resort when treating psychiatric patients	3.95 ± 0.85 (79%)	3.85 ± 0.90 (77%)	3.85 ± 0.90 (77%)	0.19
I use evidence-based practices to guide polypharmacy decisions in my psychiatric practice	3.90 ± 0.80 (78%)	3.80 ± 0.85 (76%)	3.85 ± 0.85 (77%)	0.20
I regularly consult with pharmacists or other specialists when managing complex polypharmacy cases	4.00 ± 0.85 (80%)	3.90 ± 0.85 (78%)	4.10 ± 0.70 (82%)	0.08
I believe continuing education on polypharmacy management is essential for psychiatric healthcare providers	4.35 ± 0.70 (87%)	4.25 ± 0.75 (85%)	4.30 ± 0.75 (86%)	0.15
I feel confident in educating psychiatric patients about the risks and benefits of polypharmacy	4.10 ± 0.80 (82%)	4.05 ± 0.80 (81%)	4.05 ± 0.80 (81%)	0.28
Psychiatric patients should be informed about alternative treatments to minimize polypharmacy	4.15 ± 0.80 (83%)	4.05 ± 0.85 (81%)	4.10 ± 0.80 (82%)	0.22
I involve patients and their families in decision-making regarding polypharmacy treatment plans	4.10 ± 0.75 (82%)	4.00 ± 0.75 (80%)	4.05 ± 0.75 (81%)	0.20
I provide clear explanations about each medication’s purpose and potential interactions with psychiatric patients	4.05 ± 0.85 (81%)	3.95 ± 0.85 (79%)	4.05 ± 0.85 (81%)	0.18
I encourage psychiatric patients to report any side effects or concerns related to their medications	4.35 ± 0.75 (87%)	4.30 ± 0.75 (86%)	4.30 ± 0.75 (86%)	0.25

**Note: Post**-hoc analysis: Bonferroni-adjusted comparisons identified a significant difference between psychiatrists and psychiatric nurses for Item #1 only (p < 0.05).

**Table 4** presents the perspectives of healthcare providers, including psychiatrists, nurses, and pharmacists (N = 221), on the practice of prescribing multiple psychiatric medications. The average scores, ranging from 1.95 to 4.35, indicate a generally favorable view of polypharmacy when deemed clinically necessary. Psychiatrists tend to show slightly more confidence and support for the benefits of using multiple medications, particularly in enhancing cognition, preventing relapses, and maintaining patient functionality (means 4.15 ± 4.35). However, these differences are not statistically significant (p < 0.05). Concerns about over-sedation or unnatural effects are moderate (means 3.8 ± 4.05), while worries about fatigue or sluggishness are low (means 1.95 ± 2.00), suggesting that most providers consider these side effects infrequent or less significant. Overall, the findings indicate a cautious yet generally supportive stance on polypharmacy, emphasizing the importance of careful clinical judgment, symptom management, and patient well-being, with minimal variation across different professional roles.

**Table 4:** Adapted DAI-10 attitudes toward polypharmacy by professional group (N = 221).

Statement	Psychiatrists Mean ± SD (%)	Nurses Mean ± SD (%)	Pharmacists Mean ± SD (%)	p-value
Benefits of prescribing multiple psychiatric medications outweigh the risks	3.85 ± 0.85 (77%)	3.70 ± 0.90 (74%)	3.75 ± 0.88 (75%)	0.08
Concern about patients feeling “doped up” or overly sedated	3.80 ± 0.90 (76%)	3.85 ± 0.95 (77%)	3.90 ± 0.90 (78%)	0.12
Only prescribe multiple medications when single-drug therapy is ineffective	3.90 ± 0.80 (78%)	3.80 ± 0.85 (76%)	3.85 ± 0.85 (77%)	0.10
Prescribing multiple psychiatric medications should be carefully considered due to potential unnatural effects	4.05 ± 0.85 (81%)	3.90 ± 0.87 (78%)	3.95 ± 0.88 (79%)	0.09
Prescribing multiple medications can help patients think more clearly	4.35 ± 0.70 (87%)	4.25 ± 0.75 (85%)	4.30 ± 0.75 (86%)	0.15
Prescribing multiple medications can help prevent psychiatric relapses or breakdowns	4.15 ± 0.80 (83%)	4.00 ± 0.85 (80%)	4.05 ± 0.80 (81%)	0.08
Prescribing multiple medications can help patients remain aware and function better	4.15 ± 0.80 (83%)	4.00 ± 0.85 (80%)	4.10 ± 0.80 (82%)	0.10
Confident in prescribing multiple medications when clinically appropriate	4.15 ± 0.75 (83%)	4.00 ± 0.75 (80%)	4.10 ± 0.75 (82%)	0.09
Concern that prescribing multiple medications may cause fatigue or sluggishness	2.00 ± 0.85 (40%)	1.95 ± 0.85 (39%)	2.00 ± 0.85 (40%)	0.20
Only prescribe multiple medications when necessary for managing complex symptoms	4.35 ± 0.75 (87%)	4.25 ± 0.75 (85%)	4.30 ± 0.75 (86%)	0.12

**Table 5** displays the MANOVA analysis, revealing the key factors influencing healthcare providers’ knowledge and attitudes toward psychiatric polypharmacy. Prior to regression analysis, Pearson correlation analysis showed a statistically significant positive correlation between overall knowledge and attitude toward polypharmacy (r = 0.653, p < 0.001; N = 221). Knowledge was significantly influenced by education (F = 10.506, p = 0.001, ŋp² = 0.048), confidence in managing polypharmacy (F = 27.300, p = 0.001, ŋp² = 0.115), and frequency of prescribing multiple medications (F = 33.299, p = 0.001, ŋp² = 0.137), indicating that providers with higher education levels, greater confidence, and more frequent prescribing practices demonstrated stronger knowledge. On the other hand, attitudes were significantly affected by age (F = 6.944, p = 0.009, ŋp² = 0.032), years of experience (F = 4.933, p = 0.027, ŋp² = 0.023), and the frequency of prescribing multiple medications (F = 21.826, p = 0.001, ŋp² = 0.094), suggesting that older and more experienced providers, as well as those with higher prescribing frequency, held more defined attitudes toward polypharmacy. Other variables, including gender, professional role, work setting, and psychopharmacology training, did not significantly impact knowledge or attitudes. These findings highlight that confidence and clinical practice patterns play a critical role in enhancing knowledge, whereas age and experience significantly shape healthcare providers’ attitudes toward psychiatric polypharmacy.

**Table 5:** Multivariate analysis of variance (MANOVA) of polypharmacy knowledge and attitude by participant characteristics (N = 221).

Independent Variable	Group (n)	Knowledge Mean ± SD	Attitude Mean ± SD	F	p	ŋp²
Age	<30 (n = 70)	3.85 ± 0.50	3.90 ± 0.55	Knowledge: 0.003	.958	.001
	30–39 (n = 90)	3.87 ± 0.52	4.05 ± 0.60	Attitude: 6.944	.009	.032
	≥40 (n = 61)	3.86 ± 0.48	4.10 ± 0.58			
Gender	Male (n = 120)	3.87 ± 0.50	4.00 ± 0.60	Know.: 0.917	.339	.004
	Female (n = 101)	3.85 ± 0.49	4.02 ± 0.62	Attitude: 0.270	.604	.001
Professional Role	Psychiatrist (n = 60)	4.05 ± 0.45	4.15 ± 0.50	Know.: 2.746	.099	.013
	Nurse (n = 90)	3.80 ± 0.50	3.95 ± 0.55	Attitude: 2.681	.103	.013
	Pharmacist (n = 71)	3.85 ± 0.48	4.00 ± 0.55			
Education	Bachelor (n = 120)	3.80 ± 0.50	3.95 ± 0.55	Know.: 10.506	.001	.048
	Master (n = 80)	3.95 ± 0.48	4.05 ± 0.55	Attitude: 0.434	.511	.002
	PhD (n = 21)	4.05 ± 0.45	4.10 ± 0.50			
Experience	<5 yrs (n = 80)	3.82 ± 0.50	3.90 ± 0.55	Know.: 0.156	.693	.001
	5–10 yrs (n = 90)	3.87 ± 0.48	4.00 ± 0.58	Attitude: 4.933	.027	.023
	>10 yrs (n = 51)	3.90 ± 0.50	4.05 ± 0.60			
Work Setting	Government (n = 130)	3.86 ± 0.50	4.00 ± 0.60	Know.: 0.125	.724	.001
	Private (n = 91)	3.85 ± 0.48	4.02 ± 0.58	Attitude: 0.522	.471	.002
Training	Yes (n = 120)	3.90 ± 0.48	4.05 ± 0.55	Know.: 2.551	.112	.012
	No (n = 101)	3.82 ± 0.50	3.95 ± 0.58	Attitude: 0.150	.699	.001
Confidence in Prescribing	High (n = 110)	4.15 ± 0.45	4.10 ± 0.50	Know.: 27.300	.001	.115
	Low (n = 111)	3.70 ± 0.50	3.95 ± 0.55	Attitude: 2.848	.093	.013
Prescribing Multiple Medications	Yes (n = 140)	4.20 ± 0.45	4.15 ± 0.50	Know.: 33.299	.001	137
	No (n = 81)	3.60 ± 0.50	3.85 ± 0.55	Attitude: 21.826	.001	.094

**Notes:**

• ŋp² = partial eta squared (effect size; small = .01, medium = .06, large = .14).

• Significant effects are indicated at p < .05.

• Values are presented as mean ± standard deviation (SD)

**Fig 3:**
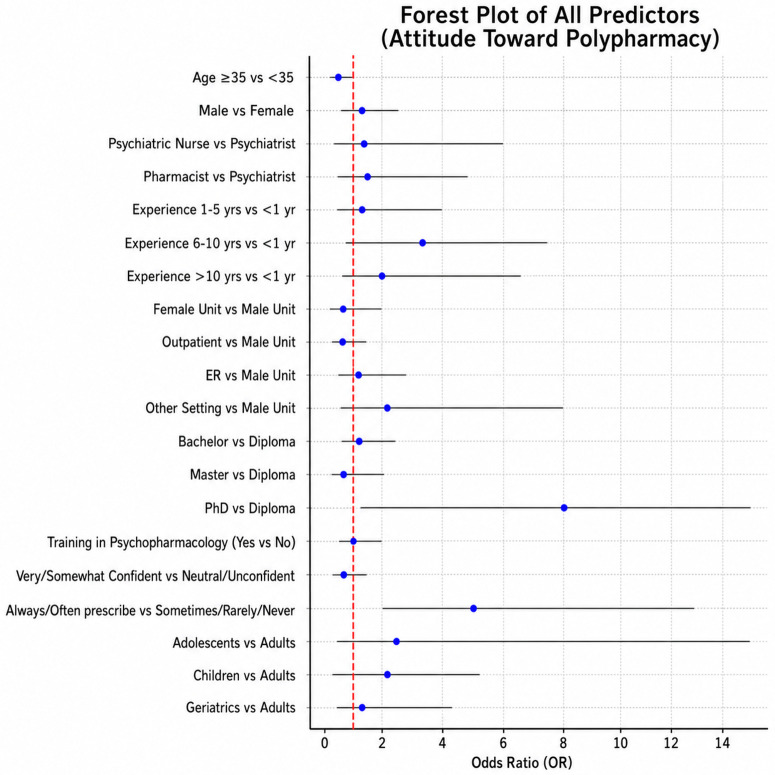
Forest plot of all predictors factors affecting the knowledge and attitudes of participants.

**Fig 3** displays the forest plot emphasizing the main factors influencing healthcare providers’ views on psychiatric polypharmacy. Being 35 years old or older was associated with notably less positive attitudes (OR = 0.36, 95% CI: 0.14–0.89), whereas possessing a PhD (OR = 8.00, 95% CI: 1.26–14.5) and routinely prescribing various medications (OR = 5.02, 95% CI: 2.04–12.5) were linked to more positive attitudes. Other elements like gender, professional position, psychiatric experience, work environment, and self-assurance were not important predictors. In general, the level of education and prescribing practices seem to be the key factors influencing attitudes.

**Fig 4:**
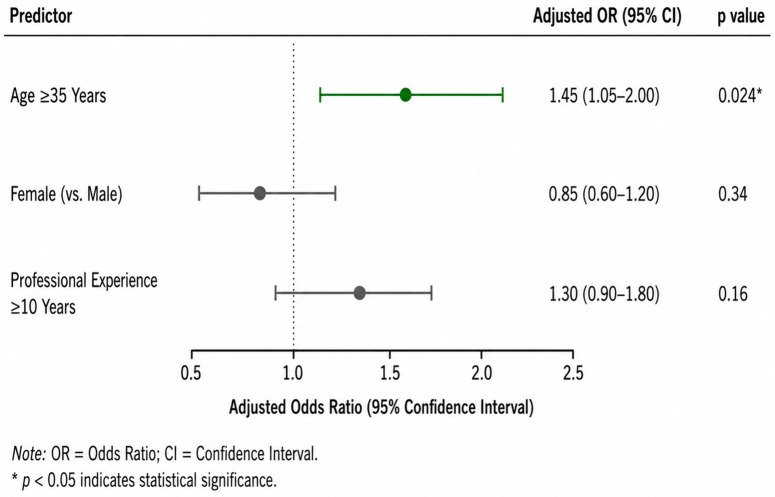
Forest plot of demographic predictors of attitudes toward polypharmacy.

**Fig 4** shows a forest plot of demographic factors associated with attitudes toward polypharmacy. The results indicate that being 35 years or older was a significant predictor of a more favorable attitude toward polypharmacy (OR = 1.45, 95% CI: 1.05–2.00), since the confidence interval did not include 1. However, female gender and having at least 10 years of professional experience were not significant predictors, as their confidence intervals included 1. Overall, these results suggest that older age was the only significant demographic factor related to a more favorable attitude toward polypharmacy.

## Results for qualitative part

**Table 6** shows that the study included 22 healthcare providers with diverse professional and demographic backgrounds. Nurses formed the largest group (41%), followed by pharmacists (32%) and psychiatrists (27%). Most participants were female (68%), with a mean age of 38.7 years (SD = 6.5). Psychiatrists were the oldest group (average age = 47.0), while pharmacists were the youngest (average age = 37.4). Nearly half (45%) had less than 10 years of experience, though psychiatrists reported significantly more experience (average = 15.2 years). The majority held a bachelor’s degree (64%), with 36% possessing advanced degrees. Nurses mainly worked at the Erada Complex for Mental Health, while psychiatrists and pharmacists were distributed across various institutions, including the Mental Health Hospital and the Psychological Health Center in Jeddah. Overall, the sample represented a well-educated, experienced group working primarily in specialized psychiatric settings.

**Table 6:** Demographic characteristics of healthcare providers (N = 22).

Variables	Total (N = 22)	Mean (SD)	Nurses (No. & %)	Psychiatrists (No & %)	Pharmacists (No & %)
Profession			**9 (41%)**	**6 (27%)**	**7 (32%)**
Gender					
Female	**15 (68%)**		**7**	**4**	**4**
Male	**7 (32%)**		**2**	**2**	**3**
Age (years)	**38.7 (6.5)**	**38.7 ± 6.5**	**38.9 ± 4.4**	**47.0 ± 4.6**	**37.4 ± 8.5**
Years of Experience (years)	**10.6 ± 3.8**	**10.6 ± 3.8**	**10.6 ± 3.8**	**15.2 ± 4.6**	**9.9 ± 6.9**
Less than 10 years	**10 (45%)**		**6**	**3**	**1**
10–20 years	**9 (41%)**		**2**	**2**	**5**
More than 20 years	**3 (14%)**		**1**	**1**	**1**
Level of Education					
Bachelor degree	**14 (64%)**		**9**	**4**	**1**
Doctorate/Advanced	**8 (36%)**		**0**	**2**	**0**
Setting of Work					
Erada Complex for Mental Health	**72 (46%)**		**7**	**0**	**0**
Al-Amal Hospital	**28 (18%)**		**2**	**0**	**0**
Mental Health Hospital, Jeddah	**20 (13%)**		**0**	**2**	**2**
Psychological Health Center, Jeddah	**31 (20%)**		**0**	**1**	**0**
Psychiatric Outpatient Clinic	**4 (3%)**		**0**	**1**	**1**

**Fig 5:**
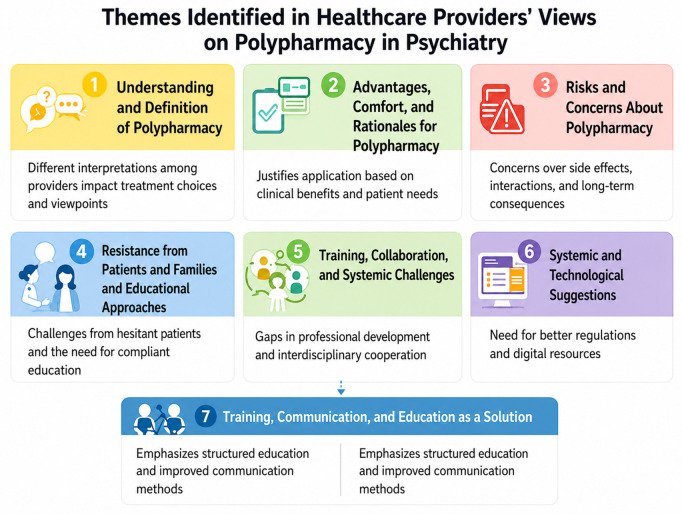
Thematic analysis of healthcare providers’ views on polypharmacy in psychiatry N = 22.

**Fig 5** illustrates the thematic analysis of healthcare providers’ views on polypharmacy in psychiatry, revealing seven main themes that provide important insights into attitudes, experiences, and challenges. ***Theme 1:*** The understanding and definition of polypharmacy showcased different interpretations among healthcare providers, impacting treatment choices and viewpoints. ***Theme 2:*** Advantages, Comfort, and Rationales for Polypharmacy illustrated how practitioners justify its application, highlighting clinical benefits and individual patient requirements. ***Theme 3:*** Risks and Concerns About Polypharmacy highlighted worries about negative effects, interactions, and long-term consequences. ***Theme 4:*** Resistance from Patients and Families and Educational Approaches examined the challenges created by patient hesitance and the importance of education in promoting compliance and comprehension. ***Theme 5:*** Training, Collaboration, and Systemic Challenges highlighted deficiencies in professional growth, communication, and interdisciplinary collaboration affecting polypharmacy practices. ***Theme 6:*** Systemic and Technological Suggestions emphasized the necessity for enhanced regulatory structures and digital resources to refine medication management. Lastly, ***Theme 7:*** Training, Communication, and Education as a Solution highlighted the significance of organized education and improved communication techniques to reduce risks and encourage safe prescribing methods. The combination of these themes provides a detailed understanding of provider viewpoints, aiding in informed actions and systemic advancements in psychiatric polypharmacy.

## Thematic analysis

This result presents the thematic analysis of interviews conducted with 9 psychiatric nurses,7 pharmacists, and 7 psychiatrists regarding their perspectives on polypharmacy. It includes main themes supported by quotes and a summary of the demographic data from participants.

### Thematic analysis of psychiatric nurses, pharmacists, and psychiatrists’ perspectives on polypharmacy

**Table:** 

Theme	Subtheme	Professional Group	Key Description	Illustrative Quote (P1–P22)
**1. Understanding and Definition of Polypharmacy**	Conceptual definition	Nurses	Polypharmacy defined as use of multiple medications for psychiatric conditions; seen as common in complex cases	“Polypharmacy refers to using multiple medications for one or more psychiatric disorders.” (P2)
	Clinical prevalence	Nurses	Recognized as frequent in complex psychiatric cases such as psychosis and comorbid conditions	“It’s quite prevalent, especially with co-occurring disorders like depression and psychosis.” (P4)
	Clinical definition	Pharmacists	Defined as concurrent use of multiple medications, especially in inpatient psychiatric settings	“We see it often in inpatient departments, especially in psychosis and schizophrenia.” (P1, P3)
	Prescribing approach	Psychiatrists	Emphasis on monotherapy first, escalation to polypharmacy when necessary	“If the patient fails in treatment, I move to polypharmacy.” (P1)
**2. Benefits and Justifications for Polypharmacy**	Clinical benefit	Nurses	Improved symptom control in treatment-resistant cases	“Combining medications helped stabilize delusional symptoms.” (P4)
	Clinical comfort	Pharmacists	Acceptable when evidence-based and monitored carefully	“I feel comfortable when polypharmacy is logical and safe.” (P7)
	Guideline-based justification	Psychiatrists	Use of APA, NICE, and local protocols to guide prescribing	“I follow APA and NICE guidelines for evidence-based prescribing.” (P6)
**3. Risks and Safety Concerns**	Adverse drug effects	Nurses	Concerns about side effects and overmedication	“Some patients develop extrapyramidal symptoms due to combinations.” (P2, P5)
	Drug interactions	Pharmacists	Risk of toxicity, interactions, and reduced adherence	“Drug interactions can lead to toxicity if not reviewed carefully.” (P6)
	Clinical complications	Psychiatrists	Concerns about long-term effects and neurological side effects	“Polypharmacy side effects may be more severe than single drugs.” (P1)
**4. Patient and Family Resistance & Education**	Patient refusal	Nurses	Resistance due to fear of side effects and overmedication	“Patients often ask why they need so many medications.” (P14)
	Counseling strategies	Pharmacists	Use of simplified explanations and tailored communication	“I simplify instructions based on patient understanding.” (P8)
	Shared decision-making	Psychiatrists	Importance of involving patients in treatment decisions	“I explain each medication and involve them in decisions.” (P5)
**5. Interprofessional Collaboration and Systemic Challenges**	Communication gaps	Nurses	Limited communication between healthcare professionals	“There is not always enough communication between staff.” (P6)
	Multidisciplinary coordination	Pharmacists	Active participation in team discussions and medication review	“I report interactions and consult physicians before dispensing.” (P7)
	Fragmented care	Psychiatrists	Lack of integration of psychologists and nurses in decision-making	“Care is often between doctor and patient only.” (P22)
**6. Training, Resources, and Technology Use**	Continuous education	All groups	Training via workshops, CME, and professional development	“We rely on journals, guidelines, and training programs.” (P5)
	Clinical resources	Pharmacists & Psychiatrists	Use of UpToDate, Maudsley Guidelines, scientific journals	“UpToDate and Maudsley guidelines are essential resources.” (P15)
	Digital tools (cautious use)	All groups	Limited but growing use of AI tools like ChatGPT for checking interactions	“ChatGPT helps, but it is not always reliable.” (P1)
**7. System-Level Recommendations for Improvement**	Electronic systems	Pharmacists	Need for digital alerts and electronic prescribing systems	“We need systems that alert drug interactions in real time.” (P3)
	Clinical pharmacy expansion	Pharmacists	Integration of clinical pharmacists in psychiatric care teams	“Every hospital should have clinical pharmacy support.” (P7)
	Education & awareness	Psychiatrists	Patient and family education improves adherence	“Once awareness is raised, resistance decreases.” (P11)

## Discussion

This study assessed the understanding and perspectives of healthcare professionals regarding polypharmacy in psychiatric environments at the Erada Complex for Mental Health Services and Addiction. The findings revealed that the participants generally possessed a high level of knowledge and held positive views on polypharmacy. Psychiatrists scored slightly higher than pharmacists and nurses, although the scores were closely grouped, indicating a ceiling effect and suggesting that the differences among professional groups were minor rather than distinctly pronounced.

The hypotheses of this study were only partially validated in this study. Specifically, H1 was not supported, as greater knowledge was anticipated to correlate with more cautious attitudes toward polypharmacy. Instead, increased knowledge was linked to more favorable attitudes, implying that a deeper understanding might boost clinicians’ confidence rather than encourage risk aversion. This outcome aligns with Bandura’s Self-Efficacy Theory [22], which posits that individuals with greater knowledge and competence feel more assured in handling complex clinical tasks. In this scenario, enhanced knowledge may bolster perceived control over pharmacological decisions rather than amplify perceived risk.

These results also resonate with the Theory of Planned Behavior [20], which suggests that attitudes are influenced by knowledge, perceived behavioral control, and professional norms. This is evident in psychiatrists’ reliance on structured clinical guidelines and pharmacists’ focus on monitoring the safety of medications. Similarly, the Health Belief Model [21] elucidates how clinicians balance perceived benefits against risks when making prescribing decisions, while Cognitive Dissonance Theory [23] offers insights into how clinicians reconcile their awareness of potential harms with the clinical necessity of polypharmacy in treatment-resistant cases. Although psychiatrists showed stronger adherence to APA and NICE guidelines, consistent with the findings of Ajayi and Arora [36], who noted that 87% of psychiatrists depend on clinical guidelines, the overall differences among the professional groups were minimal. This suggests that a shared foundational knowledge is likely shaped by institutional protocols and multidisciplinary exposure [37–40]. As highlighted by Dumba et al. [11], educational background significantly impacts psychopharmacology knowledge, while Ordak et al. [41] pointed out that clinicians may still rely on personal experience in addition to evidence-based guidelines. Pharmacists exhibited strong knowledge and actively participated in medication safety, despite having limited formal psychopharmacology certification.

Their involvement in reviewing prescriptions, identifying drug interactions, and contributing to multidisciplinary decision-making aligns with the findings of Crutzen et al. [42], who emphasized the role of pharmacists in minimizing inappropriate polypharmacy in older adults. Lawrence et al. [43] further noted that polypharmacy, particularly antipsychotic combinations, may increase adverse effects without significant additional benefit, supporting pharmacists’ cautious yet supportive approach. Notably, pharmacists endorsed polypharmacy when it was clinically justified and monitored appropriately.

Psychiatric nurses exhibited moderate knowledge and strong awareness of patient safety issues. Their relatively lower scores were attributed to limited exposure to prescribing guidelines and pharmacological decision-making, rather than a lack of competence. This aligns with previous studies that have highlighted gaps in psychopharmacology education [39,45], limited familiarity with guidelines [39], and high clinical workloads as obstacles to knowledge acquisition [45]. Qualitative findings emphasized the necessity of structured and ongoing training, especially in pharmacology and deprescribing principles. Across all professional groups, there was consistent acknowledgement of the risks associated with polypharmacy, such as adverse drug interactions, side effects, and overmedication. These findings are consistent with those of Højlund et al. [46], who noted the continued use of polypharmacy despite safety concerns. However, participants did not completely dismiss polypharmacy; instead, they supported its use when clinically justified, particularly in complex or treatment-resistant cases. This reflects a nuanced and context-dependent decision-making process that differentiates between appropriate (rational and evidence-based) and inappropriate polypharmacy’s.

Quantitative findings showed significant associations between demographic and professional characteristics and attitudes toward polypharmacy, although these should be interpreted with caution because of ceiling effects. Older healthcare professionals were less likely to express positive attitudes (OR = 0.36, 95% CI: 0.14–0.89), reflecting their greater clinical experience and risk awareness [38]. Conversely, participants with a PhD were more likely to report positive attitudes (OR = 8.00, 95% CI: 1.26–14.5), likely due to their stronger engagement with evidence-based practice, consistent with Hu et al. [47]. Frequent prescribers of multiple medications also showed more favorable attitudes (OR = 5.02, 95% CI: 2.04–12.5), suggesting that clinical exposure influences confidence and decision-making [37]. The integration of quantitative and qualitative findings provides a comprehensive understanding of polypharmacy. While quantitative results indicated high knowledge and positive attitudes, qualitative data clarified the reasons for these patterns. Psychiatrists’ adherence to guidelines explains their higher knowledge scores, pharmacists’ focus on safety monitoring contextualizes their cautious support, and nurses’ reported training needs explains their relatively lower scores. This triangulation enhances the validity of the findings and aligns with mixed-methods integration principles [42,43].

Interprofessional collaboration is crucial for ensuring safe polypharmacy. Pharmacists reported being actively engaged in multidisciplinary care; however, some nurses pointed out communication gaps among healthcare providers that could lead to inappropriate prescribing. These insights underscore the necessity of structured interdisciplinary communication and collaborative decision-making. Participants also noted an increasing dependence on digital tools, such as clinical databases and AI-assisted resources, to aid their prescription decisions. Although these tools are beneficial, concerns regarding their reliability highlight the need for a validated clinical decision support system. Implementing integrated electronic prescribing systems with real-time drug–interaction alerts could enhance medication safety and support clinical decision-making in accordance with existing evidence [44,45]. Additionally, this study improved demographic reporting by differentiating biological sex from gender, with an analysis based on sex (male/female). This method aligns with the SAGER guidelines, which advocate accurate reporting of sex-related variables to improve transparency and reproducibility in biomedical research [47].

In conclusion, healthcare professionals exhibited a high level of knowledge and generally positive attitudes toward polypharmacy in psychiatric settings. Differences among professional groups were minor and mainly reflected role-specific responsibilities rather than actual knowledge gaps. These findings are consistent with the theoretical frameworks discussed in the introduction, such as the Theory of Planned Behavior [21], Self-Efficacy Theory [23], Health Belief Model [22], and Cognitive Dissonance Theory [24], indicating that attitudes toward polypharmacy are shaped by a complex interplay of knowledge, clinical experience, perceived control, and institutional context. Future interventions should aim to strengthen multidisciplinary collaboration, improve adherence to guidelines, and provide targeted education supported by validated clinical decision-support systems to ensure safe and rational polypharmacy in older patients.

### Strengths

This study had several notable strengths. First, this is the first regional study in Saudi Arabia to examine healthcare providers’ knowledge, attitudes, and practices toward psychiatric polypharmacy, addressing a critical gap in the literature. Second, the use of a convergent mixed-methods design allowed for a comprehensive understanding by integrating quantitative and qualitative data. Third, the inclusion of psychiatrists, nurses, and pharmacists offered diverse multidisciplinary insights into polypharmacy management. Fourth, this study emphasized the essential role of psychiatric nurses, who are often central to daily medication management, highlighting their impact on patient care. Finally, analyzing the link between knowledge and attitudes provided valuable insights into how education and clinical experience shape decision-making regarding psychiatric medication use.

### Limitations

Although this study offers valuable insights, it has several limitations. First, the generally high knowledge and attitude scores suggest a possible ceiling effect, limiting the sensitivity to detect stronger differences between groups. Second, self-reported measures may introduce **social** desirability bias, particularly among health care professionals. Third, the observed intergroup differences were modest and should not be interpreted as strong categorical distinctions. Furthermore, although the Polypharmacy Knowledge Assessment Tool (PKAT) showed good internal consistency and initial construct validity through exploratory factor analysis, the lack of confirmatory factor analysis and the small pilot sample size limit the robustness of its psychometric validation. Additionally, the absence of previous regional data on psychiatric polypharmacy practices made it challenging to determine whether the observed patterns were influenced by the workplace environment or broader professional standards. Further research is necessary to verify its factor structure and relevance in larger population samples. These limitations highlight the need for future longitudinal studies to address these issues. Therefore, any conclusions about clinical behavior should be approached with caution, and multicenter studies are needed to enhance the understanding of changing clinical practices.

## Conclusion

This study provides valuable insights into healthcare professionals’ understanding and perspectives on polypharmacy within psychiatric settings at the Erada Complex for Mental Health Services and Addiction. Psychiatrists and pharmacists exhibited a relatively higher level of knowledge and more positive attitudes towards rational polypharmacy than psychiatric nurses, likely reflecting differences in their training and clinical responsibilities. Participants acknowledged that polypharmacy could be beneficial for managing treatment-resistant or complex psychiatric disorders while also recognizing the potential risks of inappropriate drug combinations, adverse effects, and communication gaps between disciplines. These findings highlight the importance of distinguishing between appropriate, evidence-based polypharmacy, and inappropriate, non-evidence-based prescribing. Rather than suggesting policy changes, the study’s results highlight the potential benefits of targeted educational programs, interprofessional collaboration, and digital decision-support tools to enhance medication safety and rational prescribing in psychiatric settings. However, given the cross-sectional nature and single-site sample of the study, these findings should be interpreted with caution and not generalized beyond similar clinical settings. Future research, including longitudinal and intervention-based studies, is recommended to explore causal relationships and evaluate strategies that support safe and evidence-based psychopharmacology.

### Recommendations and implications of the study

This study suggests that improving knowledge and interprofessional collaboration may support more informed clinical decision-making; however, further research is needed to evaluate their impact on actual prescribing practices. Therefore, recommendations are divided into three main categories: policy and institutional, educational and training, and research implications.

#### Policy and institutional recommendations.

Healthcare facilities should establish and enforce clear, evidence-based policies and guidelines for psychiatric polypharmacy that align with international standards, such as those set by the American Psychiatric Association (APA) and the National Institute for Health and Care Excellence (NICE). Hospital leaders are encouraged to regularly assess and adjust staffing levels to reduce workload pressures that may lead to overprescribing and unsafe medication practices.

Moreover, healthcare organizations should foster a supportive and non-punitive clinical environment that encourages open interdisciplinary discussions, critical evaluation of prescribing habits, and transparent reporting of medication-related issues. Such a culture of safety and collaboration can significantly improve the quality of psychopharmacological care and mitigate the risks associated with polypharmacy

#### Educational and training recommendations.

Specialized training programs in psychopharmacology should be developed and implemented for all healthcare providers—particularly psychiatric nurses—to address current knowledge gaps and promote safe medication management. Interprofessional education initiatives involving psychiatrists, pharmacists, and nurses are also essential to enhance collaboration, communication, and shared responsibility in managing polypharmacy cases.

Additionally, educational interventions should focus not only on increasing knowledge but also on improving attitudes toward polypharmacy. Emphasis should be placed on fostering critical thinking, cautious optimism, and reflective practice among healthcare providers to encourage judicious prescribing and holistic, patient-centered care.

### Research recommendations

Future research should employ longitudinal and multi-center study designs to investigate how healthcare providers’ knowledge, attitudes, and clinical practices related to polypharmacy evolve over time and across various healthcare settings. Studies are also needed to evaluate the effectiveness of structured educational programs in enhancing theoretical understanding and real-world prescribing behaviors among different professional groups.

Furthermore, research should explore the impact of organizational factors—such as hospital environment, institutional policies, and workload distribution on the safe management of psychiatric polypharmacy. Finally, demographic characteristics, including age, educational background, and clinical experience, should be examined to identify predictors of attitudes and practices, facilitating the development of tailored interventions that promote safe and effective medication use.

## Supporting information

S1 File Supplementary materials. This file contains the study instruments, statistical data file, informed consent form, facilitation letters, institutional review board approval, title page, and additional supporting documents related to the study.
(ZIP)

## Abbrevations:

APA: American Psychiatric Association
APP: Antipsychotic Polypharmacy
CI: Confidence Interval
DAI: Drug Attitude Inventory
EPS: Extrapyramidal Symptoms
HBM: Health Belief Model
HCPs: Healthcare Providers
IRB: Institutional Review Board
KAIMRC: King Abdullah International Medical Research Centre
MANOVA: Multivariate Analysis of Variance
MAPS: Multidimensional Attitudes Toward Polypharmacy Scale
NICE: National Institute for Health and Care Excellence
OR: Odds Ratio
WHO: World Health Organization
