# Supplementary material for: Psychiatric nurses versus psychiatrists and pharmacists 'knowledge on polypharmacy practices in psychiatry: An interprofessional mixed-methods exploration
Source: PLoS One. 2026 Jul 14;21(7):e0327104. doi: 10.1371/journal.pone.0327104 (PMC13367700; doi:10.1371/journal.pone.0327104)
Supplement: S1 File — This file contains the study instruments, statistical data file, informed consent form, facilitation letters, institutional review board approval, title page, and additional supporting documents related to the study. (ZIP) [file pone.0327104.s001.zip › Tools of the study.pdf]

Tools of the study

I. Demographic data of HCPs:

- **Age**:

I. _____ years

Gender:

A) Male B) Female

Professional Role:

A) Psychiatrist B) Psychiatric Nurse

D) Pharmacist F) Other (please specify): ___________

- **Years of Experience in Psychiatry**:

A) Less than 1 year B) 1-5 years

C) 6-10 years D) More than 10 years

- **Work Setting**:

A) Inpatient psychiatric unit B) Outpatient mental health clinic

C) Community home health care D) Emergency (ER)

E) Other (please specify): ___________

- **Highest Level of Education**:

A) Diploma B) Bachelor's degree

C) Master's degree D) Doctorate or PhD

E) Other (please specify): ___________

- **Additional Training in Psychopharmacology**:

A) Yes B) No

1. How confident do you feel in managing polypharmacy in psychiatric patients?

A) Very confident B) Somewhat confident C) Neutral D) Somewhat unconfident E) Very unconfident

2.How often do you prescribe multiple medications for psychiatric patients?

A) Always B) Often C) Sometimes D) Rarely E) Never

Which of the following best describes the primary population you serve?

A) Adults B) Adolescents C) Children D) Geriatrics

II. Knowledge scale of polypharmacy in psychiatry:

No.	Statements	SA	A	N	D	SD
1.	I understand the term "polypharmacy" as the use of multiple medications by a patient, particularly in psychiatric treatment.					
2.	I am aware of the potential risks associated with polypharmacy in psychiatric patients.					
3.	Polypharmacy is often necessary in psychiatric practice to manage complex mental health conditions.					
4.	I can differentiate between necessary polypharmacy and inappropriate medication use.					
5.	I am familiar with the criteria for evaluating appropriate polypharmacy in psychiatry.					
6.	Polypharmacy increases the likelihood of drug-drug interactions in psychiatric patients.					
7.	I understand the potential for increased side effects with polypharmacy in psychiatric treatment.					
8.	Patients on polypharmacy are more likely to experience medication non-adherence.					
9.	Polypharmacy can contribute to cognitive impairment in psychiatric patients.					
10.	I am aware of how polypharmacy can exacerbate comorbid physical health conditions in psychiatric patients					
11.	Regular medication reviews are crucial for managing polypharmacy in psychiatric care.					
12.	I know how to assess the effectiveness and necessity of each medication in a patient's polypharmacy regimen.					
13.	I am familiar with deprescribing strategies to reduce polypharmacy when appropriate.					
14.	I understand the importance of involving patients in discussions about their polypharmacy treatment plans.					
15.	I monitor for signs of adverse drug reactions or toxicity in psychiatric patients on multiple medications.					
16.	I am aware of clinical guidelines that provide recommendations for managing polypharmacy in psychiatry.					
17.	Polypharmacy should always be considered a last resort when treating psychiatric patients.					
18.	I use evidence-based practices to guide polypharmacy decisions in my psychiatric practice.					

19.	I regularly consult with pharmacists or other specialists when managing complex polypharmacy cases.					
20.	I believe continuing education on polypharmacy management is essential for psychiatric healthcare providers					
21.	I feel confident in educating psychiatric patients about the risks and benefits of polypharmacy.					
22.	Psychiatric patients should be informed about alternative treatments to minimize polypharmacy.					
23.	I involve patients and their families in decision-making regarding polypharmacy treatment plans.					
24.	I provide clear explanations about each medication's purpose and potential interactions to psychiatric patients.					
25.	I encourage psychiatric patients to report any side effects or concerns related to their medications.					

III. Adapted DAI-10 tailored to healthcare providers' attitudes toward prescribing polypharmacy in psychiatric settings:

No.	Statement	SA	A	N	D	SD
**1.**	For my patients, the benefits of prescribing multiple psychiatric medications outweigh the risks.					
**2.**	I am concerned that prescribing multiple medications may make patients feel "doped up" or overly sedated					
**3.**	I only prescribe multiple medications when single-drug therapy is ineffective.					
**4.**	I believe that prescribing multiple psychiatric medications should be carefully considered due to the potential unnatural effects on the patient's mind and body.					
**5.**	I believe that prescribing multiple medications can help patients think more clearly.					
**6.**	I believe that prescribing multiple medications can help prevent psychiatric relapses or breakdowns.					
**7.**	I believe prescribing multiple medications can help patients remain more aware and function better in daily life					
**8.**	I am confident in my choice to prescribe multiple medications when clinically appropriate for my patients					
**9.**	I am concerned that prescribing multiple medications may cause fatigue and sluggishness in my patients.					
**10.**	I only prescribe multiple psychiatric medications when they are necessary for managing complex symptoms.					

Qualitative data:
Semi structured interview questions guide for HPCs opinions and perspectives related to polypharmacy:

1. What roles have you held? How long have you worked in this field?
2. What does polypharmacy mean to you, and how prevalent do you think it is in your practice?

3. Do you feel comfortable prescribing polypharmacy? Why or why not?
4. Are there specific conditions, patient needs, or guidelines that impact your decisions?
5. In your opinion, what are the potential benefits of polypharmacy in treating psychiatric disorders? Provide examples:
6. What risks do you associate with polypharmacy? Have you encountered adverse effects in your practice?
7. What strategies do you use to ensure patients understand their treatment plans?
8. Have you encountered patients who are resistant to polypharmacy? If so, how do you address their concerns?
9. Have you received any training on this topic? What additional resources would you find helpful?
10. Are there specific guidelines you follow? How do they impact your prescribing behavior?
11. How do you collaborate with other healthcare providers when managing patients on multiple medications?
12. What improvements do you think could be made in the healthcare system to better support HCPs in managing polypharmacy?
13. How do you think this challenge can be addressed?
14. Any additional thoughts, concerns, or insights you haven’t covered?
