# Supplementary material for: Psychiatric nurses versus psychiatrists and pharmacists 'knowledge on polypharmacy practices in psychiatry: An interprofessional mixed-methods exploration
Source: PLoS One. 2026 Jul 14;21(7):e0327104. doi: 10.1371/journal.pone.0327104 (PMC13367700; doi:10.1371/journal.pone.0327104)
Supplement: S1 File — This file contains the study instruments, statistical data file, informed consent form, facilitation letters, institutional review board approval, title page, and additional supporting documents related to the study. (ZIP) [file pone.0327104.s001.zip › updated Title Page.docx]

**" Psychiatric Nurses Versus Psychiatrists and Pharmacists 'Knowledge on Polypharmacy Practices in Psychiatry: An Interprofessional Mixed-Methods Exploration"**

**Amal I. Khalil**

King Abdullah International Medical Research Center, Jeddah, Saudi Arabia

King Saud bin Abdulaziz University for Health Sciences, College of Nursing, Jeddah, KSA

Ministry of National Guard health Affairs (MNGHA)

Menoufyia University, Faculty of Nursing, Egypt

ORCID: <https://orcid.org/0000-0001-8419-2289>,

Web of Science Researcher ID: HGD-9271-2022

E-mail: khalila@ksau-hs.edu.sa and [amalkhalil34@yahoo.com](mailto:amalkhalil34@yahoo.com)

**Alhanouf A. Almuhalbidi**

King Abdullah International Medical Research Center, Jeddah, Saudi Arabia

King Saud bin Abdulaziz University for Health Sciences, College of Nursing, Jeddah, KSA

Ministry of National Guard health Affairs (MNGHA)

**Email:** [almuhalbidi20507@ksau-hs.edu.sa](mailto:almuhalbidi20507@ksau-hs.edu.sa)

**Reema T. Almutairi**

King Abdullah International Medical Research Center, Jeddah, Saudi Arabia

King Saud bin Abdulaziz University for Health Sciences, College of Nursing, Jeddah, KSA

Ministry of National Guard health Affairs (MNGHA)

**Email:** **almutairi20577@ksau-hs.edu.sa**

**Shahad S. Almutairi**

King Abdullah International Medical Research Center, Jeddah, Saudi Arabia

King Saud bin Abdulaziz University for Health Sciences, College of Nursing, Jeddah, KSA

Ministry of National Guard health Affairs (MNGHA)

**Email:** **almutairi20598@ksau-hs.edu.sa**

**Hatun H. Alansari**

King Abdullah International Medical Research Center, Jeddah, Saudi Arabia

King Saud bin Abdulaziz University for Health Sciences, College of Nursing, Jeddah, KSA

Ministry of National Guard health Affairs (MNGHA)

**Email:** **alansari20647@ksau-hs.edu.sa**

**Atheer S. Almarri**

King Abdullah International Medical Research Center, Jeddah, Saudi Arabia

King Saud bin Abdulaziz University for Health Sciences, College of Nursing, Jeddah, KSA

Ministry of National Guard health Affairs (MNGHA)

**Email:** **almarri20518@ksau-hs.edu.sa**

**Shaima K. Alzahrani**

King Abdullah International Medical Research Center, Jeddah, Saudi Arabia

King Saud bin Abdulaziz University for Health Sciences, College of Nursing, Jeddah, KSA

Ministry of National Guard health Affairs (MNGHA)

**Email:** [**alzahrani20663@ksau-hs.edu.sa**](mailto:alzahrani20663@ksau-hs.edu.sa)

**Joud s. Alzahrani**

King Abdullah International Medical Research Center, Jeddah, Saudi Arabia

King Saud bin Abdulaziz University for Health Sciences, College of Nursing, Jeddah, KSA

Ministry of National Guard health Affairs (MNGHA)

**Email:** **alzahrani20541@ksau-hs.edu.sa**

**Corresponding author:**

**Amal I. Khalil**

King Abdullah International Medical Research Center, Jeddah, Saudi Arabia

King Saud bin Abdulaziz University for Health Sciences, College of Nursing, Jeddah, KSA

Ministry of National Guard health Affairs (MNGHA)

Menoufyia University, Faculty of Nursing, Egypt

ORCID: <https://orcid.org/0000-0001-8419-2289>,

Web of Science Researcher ID: HGD-9271-2022

E-mail: khalila@ksau-hs.edu.sa and [amalkhalil34@yahoo.com](mailto:amalkhalil34@yahoo.com)

**Author Contribution Update**

Joud S. Alzahrani
Affiliation: King Abdullah International Medical Research Center, Jeddah, Saudi Arabia; King Saud bin Abdulaziz University for Health Sciences, College of Nursing, Jeddah, Saudi Arabia; Ministry of National Guard Health Affairs (MNGHA), Jeddah, Saudi Arabia.
Email: alzahrani20541@ksau-hs.edu.sa.

Contribution: Joud S. Alzahrani contributed to data collection, data curation, literature review, manuscript drafting, and critical revision of the manuscript. She reviewed and approved the final version of the manuscript.
