# Supplementary material for: Psychiatric nurses versus psychiatrists and pharmacists 'knowledge on polypharmacy practices in psychiatry: An interprofessional mixed-methods exploration
Source: PLoS One. 2026 Jul 14;21(7):e0327104. doi: 10.1371/journal.pone.0327104 (PMC13367700; doi:10.1371/journal.pone.0327104)
Supplement: S1 File — This file contains the study instruments, statistical data file, informed consent form, facilitation letters, institutional review board approval, title page, and additional supporting documents related to the study. (ZIP) [file pone.0327104.s001.zip › SUPPORTING INFORMATION.pdf]

SUPPORTING INFORMATION

Overview

This Supporting Information document provides all supplementary materials used in this study to ensure transparency, reproducibility, and methodological rigor.

The study adheres to:

- **STROBE guidelines** for observational components
- **COREQ checklist** for qualitative research
- **CONSORT principles** for reporting intervention components where applicable

S1 File. Arabic Version of the MAPS Instrument and Related Tools

This file contains the full Arabic versions of all study instruments used for healthcare providers (HCPs), including:

Section I: Demographic Data

- Age
- Gender
- Professional role
- Years of experience in psychiatry
- Work setting
- Educational level
- Additional training in psychopharmacology

Section II: Knowledge Scale of Polypharmacy in Psychiatry

- 25 items assessing knowledge of:
    - Definition and concepts of polypharmacy
    - Risks (e.g., drug interactions, side effects)
    - Clinical decision-making
    - Deprescribing strategies
    - Patient education and engagement
- Response format: 5-point Likert scale

Section III: Adapted DAI-10 (Arabic Version)

- Modified to assess healthcare providers' attitudes toward polypharmacy
- Covers:
    - Risk–benefit perceptions

    - Clinical judgment
    - Confidence in prescribing
    - Concerns about adverse effects

Section IV: Qualitative Interview Guide (Arabic)

- Semi-structured interview questions exploring:
    - Definitions and perceptions of polypharmacy
    - Clinical experiences
    - Decision-making processes
    - Barriers and facilitators
    - Interprofessional collaboration
    - System-level challenges

Translation and Cultural Adaptation Process

The Arabic version was developed using a rigorous methodology:

1. Forward translation by bilingual experts
2. Backward translation
3. Expert panel review (psychiatry and nursing specialists)
4. Pilot testing with healthcare providers
5. Refinement for clarity and cultural relevance

S2 File. English Version of Study Instruments

This file includes the original English versions of all tools used in the study:

Section I: Demographic Questionnaire

- Captures participant characteristics and professional background

Section II: Knowledge Scale

- 25-item validated scale assessing knowledge of polypharmacy in psychiatry

Section III: Adapted DAI-10

- Measures attitudes toward prescribing multiple psychiatric medications

Section IV: Qualitative Interview Guide

- Semi-structured questions aligned with study objectives
- Designed to explore experiences, perceptions, and clinical practices
