# Supplementary material for: Psychiatric nurses versus psychiatrists and pharmacists 'knowledge on polypharmacy practices in psychiatry: An interprofessional mixed-methods exploration
Source: PLoS One. 2026 Jul 14;21(7):e0327104. doi: 10.1371/journal.pone.0327104 (PMC13367700; doi:10.1371/journal.pone.0327104)
Supplement: S1 File — This file contains the study instruments, statistical data file, informed consent form, facilitation letters, institutional review board approval, title page, and additional supporting documents related to the study. (ZIP) [file pone.0327104.s001.zip › Facilitation_Letter_2.pdf]

King Saud bin Abdulaziz University for Health Sciences

National Guard Health Affairs

Jeddah, Saudi Arabia

Date: 14 January 2025

Facilitation Letter for Research Conduct

To:

His Excellency, the Concerned Authority

Greetings,

This letter is issued to facilitate the task of the researcher in conducting an approved scientific research study at your esteemed institution.

The research has received the necessary administrative and ethical approvals from the relevant authorities. Accordingly, we kindly request your cooperation and support by allowing the researcher to carry out the required data collection procedures, in accordance with institutional regulations and approved research policies.

The researcher is committed to adhering to all ethical principles, data confidentiality requirements, and non-disclosure agreements applicable during the research period.

We highly appreciate your cooperation and support of scientific research.

Yours sincerely,

Authorized Signatory

Academic Affairs, Training and Research
