# Supplementary material for: Psychiatric nurses versus psychiatrists and pharmacists 'knowledge on polypharmacy practices in psychiatry: An interprofessional mixed-methods exploration
Source: PLoS One. 2026 Jul 14;21(7):e0327104. doi: 10.1371/journal.pone.0327104 (PMC13367700; doi:10.1371/journal.pone.0327104)
Supplement: S1 File — This file contains the study instruments, statistical data file, informed consent form, facilitation letters, institutional review board approval, title page, and additional supporting documents related to the study. (ZIP) [file pone.0327104.s001.zip › Facilitation_Letter 3.pdf]

King Saud bin Abdulaziz University for Health Sciences

National Guard Health Affairs

Jeddah, Saudi Arabia

Date: 12 March 2025

Ethical Approval No.: A02107

Facilitation Letter for Research Conduct

To:

His Excellency, the General Supervisor

Eradah Complex for Mental Health Services

Greetings,

We would like to inform you that the research entitled:

“Understanding Healthcare Providers' Knowledge and Attitudes Toward Polypharmacy in Psychiatry Versus Psychiatric Patients' Perspectives on Medication Management: A Mixed-Methods Study”

has obtained ethical approval from the Local Medical Research Ethics Committee, Health Affairs, under approval number A02107, dated 12/03/2025. The approval has been granted to the Principal Investigator, Dr. Amal Khalil.

Accordingly, we kindly request your permission to conduct the above-mentioned research at your esteemed facility and to facilitate the researcher’s task by directing the concerned departments to cooperate in the data collection process, in accordance with the applicable regulations and procedures approved by your Research Administration.

Please note that the research team is committed to full compliance with the Data Sharing Agreement and the Non-Disclosure Agreement, and to adhering strictly to all approved ethical and administrative requirements.

Study Duration: Six (6) months

Principal Investigator: Dr. Amal Khalil

Affiliation: King Saud bin Abdulaziz University for Health Sciences

Specialty: Nursing

Contact Number: 0595138896

Yours faithfully,

Dr. Ahmed Ezzeldin Khalaf

Vice Executive President for Academic Affairs, Training, and Research

Jeddah First Health Cluster
