# Supplementary material for: Psychiatric nurses versus psychiatrists and pharmacists 'knowledge on polypharmacy practices in psychiatry: An interprofessional mixed-methods exploration
Source: PLoS One. 2026 Jul 14;21(7):e0327104. doi: 10.1371/journal.pone.0327104 (PMC13367700; doi:10.1371/journal.pone.0327104)
Supplement: S1 File — This file contains the study instruments, statistical data file, informed consent form, facilitation letters, institutional review board approval, title page, and additional supporting documents related to the study. (ZIP) [file pone.0327104.s001.zip › facilitation letter 1 .pdf]

تسهيل مهمة باحث

سعادة المشرف العام على مجمع إرادة للخدمات النفسية المحترم

نفيدكم علما بان البحث بعنوان
(Understanding Healthcare Providers' Knowledge and Attitudes Toward Polypharmacy in Psychiatry Versus Psychiatric Patients' Perspectives on Medication Management: A Mixed-Methods Study)
للباحثة (د/ أمل خليل) حصل على الموافقة الأخلاقية من اللجنة المحلية للبحوث الطبية بالشؤون الصحية برقم (A02107) وتاريخ (12/03/2025)
نأمل منكم الإيعاز لمن يلزم بتسهيل مهمة الباحثة والسماح لها بإجراء البحث في منشأتكم بعد استيفاء الضوابط اللازمة لجمع البيانات المعدة من قبل إدارة البحوث بالمنشأة لديكم
مع ضرورة تقيد الباحثين بالشروط التي تم الإقرار عليها في اتفاقية مشاركة البيانات واتفاقية عدم الإفصاح

Understanding Healthcare Providers' Knowledge and Attitudes Toward Polypharmacy in Psychiatry Versus Psychiatric Patients' Perspectives on Medication Management: A Mixed-Methods Study	عنوان البحث
A02107	رقم الموافقة الأخلاقية
12/03/2025	تاريخ الموافقة الاخلاقية
6 أشهر	مدة البحث
د/ أمل خليل	الباحث الرئيسي
جامعة الملك سعود الصحية بالحرس الوطني	جهة عمل الباحث الرئيسي
تمريض	التخصص
0595138896	بيانات التواصل مع الباحث

نائب الرئيس التنفيذي للشئون الاكاديمية والتدريب
والبحوث لتجمع جدة الصحي الأول
Zainab
د. زينب أحمد عزالدين خلف
