# Supplementary material for: Psychiatric nurses versus psychiatrists and pharmacists 'knowledge on polypharmacy practices in psychiatry: An interprofessional mixed-methods exploration
Source: PLoS One. 2026 Jul 14;21(7):e0327104. doi: 10.1371/journal.pone.0327104 (PMC13367700; doi:10.1371/journal.pone.0327104)
Supplement: S1 File — This file contains the study instruments, statistical data file, informed consent form, facilitation letters, institutional review board approval, title page, and additional supporting documents related to the study. (ZIP) [file pone.0327104.s001.zip › IRB approval_a4fbe.pdf]

وزارة الصحة
Ministry of Health

الإدارة: إدارة التغيير والتحول بفرع وزارة الصحة بجده (قسم البحوث)
الموضوع: الموافقة الاخلاقية

سعادة مدير إدارة البحوث والدراسات بالتجمع الصحي الثاني المحترم
السلام عليكم ورحمة الله وبركاته...
نفيدكم بأن الباحث الموضحة بياناته في الجدول التالي قد استوفى جميع الشروط التي تنص عليها اللائحة التنفيذية لأخلاقيات البحوث والصادرة من مدينة الملك عبدالعزيز للعلوم والتقنية ودليل الممارسة الأخلاقية الجيدة وقد تمت اجازته من قبل اللجنة المحلية لأخلاقيات البحوث بفرع الوزارة بجده (الموافقة المبدئية) لاستكمال خطوات البحث وجمع البيانات .

**أسم الباحث الرئيسي**	امل خليل
**عنوان البحث**	**Understanding Healthcare Providers' Knowledge and Attitudes Toward Polypharmacy in Psychiatry Versus Psychiatric Patients' Perspectives on Medication Management: A Mixed-Methods Study**
**المنشات المراد اجراء البحث فيها**	مجمع إرادة للصحة النفسية- خدمات ارادة
**رقم الموافقة**	**A02115**
**مدة البحث**	**٦ أشهر فقط**
**التاريخ**	**01/01/2025**
**رقم التواصل مع الباحث**	**٠٥٩٥١٣٨٨٩٦**

ملاحظة مهمه:
1. ضرورة تقيد الباحث بالشروط التي تم الإقرار عليها في خطاب الموافقة الأخلاقية
2. تزويد اللجنة المحلية لأخلاقيات البحوث بفرع وزارة الصحة بجدة بنتائج البحث والنسخة النهائية وذلك لاستكمال منح الباحث الموافقة النهائية (للنشر)

وتفضلوا بقبول أطيب تحياتي

رئيس اللجنة المحلية لأخلاقيات البحوث بفرع وزارة الصحة بجده

د / علا أكرم عبد الرشيد

مديرية الشئون الصحية بمحافظة جدة
وزارة الصحة
Ministry of Health
H-02-J-002
Institutional Review Board
اللجنة المحلية لأخلاقيات البحث العلمي

Scanned with CamScanner

Kingdom of Saudi Arabia

Ministry of Health

المملكة العربية السعودية
وزارة الصحة
مديرية الشؤون الصحية بمحافظة جدة

اللجنة المحلية لأخلاقيات البحوث بصحة جدة

Institutional Review Board

National Registration Number with NCBE-KACST, KSA: (H-02-J-002)

Approval Letter

Date:01/01/2025
IRB Log No: A02115
Category of Approval: Expedited

Dear Researcher

The IRB is pleased to inform you that your study mentioned below has been reviewed and approved. This letter gives you an ethical clearance to implement your study according to the approved documents and you still need to obtain administrative approval from the site/s where the study will be conducted.

**Protocol Title**	**Understanding Healthcare Providers' Knowledge and Attitudes Toward Polypharmacy in Psychiatry Versus Psychiatric Patients' Perspectives on Medication Management: A Mixed-Methods Study**		
**PI Name**	**Amal Khalil**	**PI ID**	**2278005554**
**PI Affiliation**	**Non-MOH**	**PI E-mail**	**khalila@ksau-hs.edu.sa**
**IRB Approved Documents (attached)**	☒ Research proposal<br>☒ Consent form<br>☒ Data collection tool<br>☐ Others, ______________		

**Co-Investigators**	#	Name	ID
	#	Alhanouf Abdullah Almuhalbidi	1119297909
		Atheer Saeed Almarri	1115303164
		Hatun Hamid Alansari	1121325029
		Joud salehAlzahrani	1170892929
		Reema Tariq Almutairi	1125113801
		Shahad Salman Almutairi	1118982584
		Shaima Khaled Alzahrani	1116364553

**Study Site and Sample**	#	Site	Sample size
	#	Eradah complex for mental health-Eradah services	
			Total sample = # participants

IRB E-mail: IRB-JEDDAH@MOH.GOV.SA

Scanned with CamScanner

Kingdom of Saudi Arabia

Ministry of Health

المملكة العربية السعودية
وزارة الصحة
مديرية الشؤون الصحية بمحافظة جدة

اللجنة المحلية لأخلاقيات البحوث بصحة جدة

Institutional Review Board

National Registration Number with NCBE-KACST, KSA: (H-02-J-002)

Approval Conditions:

1. The approval is valid for **one year** from the date of this letter.
2. If the research is not completed within the validation period, the PI will be required to apply for an extension from the IRB, two months before the expiry of the approval.
3. Abide by the rules and regulations of the Government of Saudi Arabia, NCBE, MOH and the IHC-GCP guidelines.
4. The research team should follow the IRB approved study documents, unless amendment(s) are requested and approved by the IRB.
5. All researchers are required to have a valid research ethics certificate on protecting human research participants.
6. The research team is not allowed to disclose personally identifiable data of the participants to any other party.
7. The PI is required to keep the study data securely for at least three years after the completion of the study.
8. The collected data should only be used for this research.
9. It is required to collect three copies of informed consent forms (unless waived) as follow:
   I. One copy to be kept with the PI
   II. One copy to be kept with the study participant
   III. One copy for the IRB committee OR to be kept in the participant file in case of clinical research
10. The PI is required to submit a progress report **every six months**
11. The PI must ensure adequate close-out of the study.
12. Publication by any means is not allowed except after getting an approval letter from the IRB and MOH research department.

Sincerely yours,

Signature

Dr. Ola Akram Abdulrashid ,MD

Chairman of Jeddah IRB, MOH, KSA.

IRB E-mail: IRB-JEDDAH@MOH.GOV.SA

Scanned with CamScanner
