# Supplementary material for: Psychiatric nurses versus psychiatrists and pharmacists 'knowledge on polypharmacy practices in psychiatry: An interprofessional mixed-methods exploration
Source: PLoS One. 2026 Jul 14;21(7):e0327104. doi: 10.1371/journal.pone.0327104 (PMC13367700; doi:10.1371/journal.pone.0327104)
Supplement: S1 File — This file contains the study instruments, statistical data file, informed consent form, facilitation letters, institutional review board approval, title page, and additional supporting documents related to the study. (ZIP) [file pone.0327104.s001.zip › Cross_sectional_inform_consent_form copy 2.pdf]

Kingdom of Saudi Arabia
Ministry of National Guard - Health Affairs

المملكة العربية السعودية
وزارة الحرس الوطني – الشؤون الصحية

Informed Consent for Cross Sectional Surveys

إقرار موافقة للمشاركة بدراسة مقطعية

Study No. : Psychiatric Nurses Versus Psychiatrists and Pharmacists 'Knowledge on Polypharmacy Practices in Psychiatry: An "Interprofessional Mixed-Methods Exploration"

Principal Investigator : Dr. Amal Ebraheem Khalil

You are requested to participate in research that will be supervised by (Dr.Amal Ebraheem Khalil) in (Jeddah-Saudi Arabia).	أنت مدعو للانضمام طواعية لدارسة بحثية سوف يشرف عليها (د.امل إبراهيم خليل) في (جدة-المملكة العربية السعودية)
The aim of this study is to assess healthcare providers' knowledge and attitudes toward polypharmacy in psychiatric practices and exploring professional perspectives and opinions regarding polypharmacy)	هذه الدراسة تهدف إلى تقييم معرفة ومفاهيم مقدمي الرعاية الصحية تجاه تعدد الأدوية في الممارسات النفسية، واستكشاف وجهات نظر وارائهم بشأن تعدد الأدوية.
In psychiatric care, understanding healthcare providers' knowledge and attitudes toward polypharmacy is essential for safe and effective prescribing, minimizing risks like drug interactions. Additionally, considering patients' perspectives fosters collaboration and improves adherence to treatment. By integrating these insights, medication management and overall patient outcomes can be enhanced, highlighting the significance of evaluating both providers' knowledge and attitudes alongside patients' awareness and opinions on polypharmacy.	في الرعاية النفسية، من الضروري فهم معرفة مقدمي الرعاية الصحية تجاه تعدد الأدوية، حيث يساعد ذلك في ضمان وصف آمن وفعال للأدوية ويقلل من المخاطر مثل التفاعلات الدوائية. كما أن استكشاف آراء المرضى يعزز التعاون ويزيد من التزامهم بالعلاج. من خلال دمج هذه الآراء، يمكن تحسين إدارة الأدوية وتحقيق نتائج أفضل للمرضى، مما يُبرز أهمية تقييم وجهات نظر كل من مقدمي الرعاية والمرضى حول تعدد الأدوية.
Your participation is voluntary, and you have the right to not complete this survey without giving any reason and this will not affect your current or future medical care in MNG-HA.	إن مشاركتك في هذه الدراسة طوعية ولك الحق التام في عدم قبول تعبئة الاستمارة أو الانسحاب في أي وقت تشاء بدون ابداء الاسباب ولن يؤثر ذلك على العناية الطبية المقدمة لك حالياً أو في المستقبل في الشؤون الصحية بوزارة الحرس الوطني.
You do not have to sign this information sheet only you can choose to agree/disagree; your acceptance to complete the survey will be interpreted as your informed consent to participate.	لا يجب عليك التوقيع على ورقة المعلومات هذه ، فقط عليك الاختيار موافق / غير موافق فمجرد قبولك تعبئة هذا الاستبيان يعتبر بمثابة إقرارك بالموافقة على المشاركة في هذا البحث .
Your responses will be kept anonymous. However, whenever one works with email/the internet there is always the risk of compromising privacy, confidentiality, and/or anonymity. Despite this possibility, the risks to your physical, emotional, social, professional, or financial well-being are considered to be 'less than minimal'.	ستبقى الردود على الأسئلة سرية ومع ذلك ، فإن العمل عن طريق البريد الالكتروني والانترنت يبقى هناك احتمال الاختراق خصوصية البيانات وسرية المعلومات ولكن بالرغم من هذه الاحتمالية تبقى الاخطار البدنية والعاطفية والاجتماعية والمهنية والمالية المترتبة عليك ضمن الحد الادنى من الخطورة.
If you have any questions about the research, please contact (Dr.Amal Ebraheem Khalil) (College of Nursing)(khalila@ksau-hs.edu.sa)(0595138896)	إذا كان لديك أي اسئلة حول هذا البحث ، يرجى الاتصال (د.امل إبراهيم خليل)(كلية التمريض، جامعة الملك سعود بن عبدالعزيز للعلوم الصحية)(khalila@ksau-hs.edu.sa)(0595138896)
In case you have any enquiries related to your rights as a research subject you can contact the Institutional Review Board on Tel 8011111 Ext. 14572.	في حال كان لديك الاستفسارات المتعلقة بحقوقك كموضوع بحث يمكنك الاتصال بمجلس المراجعة المؤسسية على هاتف 8011111 تحويلة 14572
☐ Agree to participate<br>☐ Disagree to participate	موافق على المشاركة ☐<br>غير موافق على المشاركة ☐

This information shall not be used, disclosed, or published without written approval from King Abdullah International Medical Research Center

Version No. :

Version Date:
